# Survival comparison between postoperative and preoperative radiotherapy for stage I–III non-inflammatory breast cancer

**DOI:** 10.1038/s41598-022-18251-3

**Published:** 2022-08-22

**Authors:** Yuxi Zhang, Zhipeng Xu, Hui Chen, Xinchen Sun, Zhaoyue Zhang

**Affiliations:** 1 The First School of Clinical Medicine, Nanjing Medical University, Nanjing, China; 2 Department of Urology, Affiliated Zhongda Hospital of Southeast University, Nanjing, Jiangsu China; 3 Department of Radiation Oncology, Jiangsu Province Hospital, Nanjing, China

**Keywords:** Cancer, Breast cancer

## Abstract

To compare the survival benefit between preoperative and postoperative radiotherapy for stage I–III non-inflammatory breast cancer patients, we conducted a retrospective cohort study using surveillance, epidemiology and end results databases. Our study recruited patients who had been diagnosed with stage I–III breast cancer and underwent surgery and radiotherapy. The overall survival was calculated by Kaplan–Meier method. Cox risk model was used to determine the impact of radiotherapy according to stage, molecular subtype and other risk factors. Propensity score matching was used to balance measurable confounding factors. Of all the 411,279 enrolled patients varying from 1975 to 2016, 1712 patients received preoperative radiotherapy, and 409,567 patients received postoperative radiotherapy. Compared with the postoperative radiotherapy group, the preoperative radiotherapy group showed significantly higher risks of overall mortality and breast cancer-specific mortality. Survival differences in treatment sequences were correlated with stage, molecular subtypes and other risk factors. According to the results of this study, preoperative radiotherapy did not show a survival advantage, and postoperative radiotherapy is still the primary treatment. However, preoperative radiotherapy also has some theoretical advantages, such as phase reduction and recurrence reduction. Therefore, it is still worthy of further exploration.

## Introduction

Recently, the International Agency for Research on Cancer of the World Health Organization released the latest global cancer burden data in 2020. The incidence rate of breast cancer is the world's first, with a mortality rate of fifth. The incidence and mortality of breast cancer in women were both the first. Breast cancer has officially replaced lung cancer and becomes the most prominent cancer in the world. The number of cancer deaths in China ranks first in the world. In 2020, there will be 4.57 million new cancer cases in China, and the incidence of breast cancer is the highest in the world. One of the fundamental reasons for the increase in breast cancer cases is the constantly changing risk factors of breast cancer, such as delayed birth and fewer births. This is most evident in countries with socioeconomic transition, overweight and obesity. Moreover, lack of exercise has led to an increase in the incidence rate of breast cancer worldwide.

This clinical condition is usually associated with an increased risk of local recurrence, distant metastasis, reduced quality of life and overall survival. The standard care for early patients is mastectomy or mastectomy plus lymph node sampling, followed by adjuvant radiotherapy (RT) on the tumor bed or the whole breast as directed^1^, which has been shown to reduce the risk of recurrence^2^. Standard treatment for locally advanced breast cancer is always multimodalities, including chemotherapy and/or hormone therapy, surgery and RT for systemic^3,4^. For patients with T3 or T4 disease or positive lymph nodes, adjuvant RT for the chest wall and any at-risk axillary sites should be considered after total mastectomy^1^. When the non-operative standard prevails, RT can be used for tumor descent. In this case, RT can significantly reduce the recurrence rate of the ipsilateral breast and the specific mortality rate of breast cancer^5,6^. Recently, the hypothesis of anticipating RT before surgery has been considered interesting and has attracted more and more attention. The potential advantages of preoperative RT for breast cancer include accurate tumor location and better target area delineation, and may reduce tumor stage with the increase of breast conserving surgery rate. In addition, by avoiding the irradiation of tissue expander, implant or autologous tissue flap, patients who need breast reconstruction after mastectomy are expected to improve the cosmetic effect of surgery or reduce reconstruction complications^7^. In addition, preoperative RT strategy can overcome possible technical problems. Treatment planning challenges after reconstruction surgery^8–10^. preoperative RT may also increase the total pathological complete remission rate, which may represent another step toward precision medicine, allowing breast cancer RT to be tailored for each patient and stratifying the risk of receiving postoperative therapy. If RT is applied before surgery, it can be used as a tool for treatment stratification and even as a predictor of degrading treatment in the case of complete pathological response^11–13^. The increased use of hypofractionation and accelerated partial breast irradiation has created a new era of breast RT^14^, which may alleviate some problems, such as delaying planned chemotherapy due to the extension of preoperative RT course. Given the scarcity of literature, a population-based analysis of the long-term impact of preoperative RT has not been carried out. We designed a study to assess the overall survival and cancer specific survival in patients with non-inflammatory non-metastatic breast cancer who received postoperative or preoperative RT.

## Materials and methods

### Materials

The surveillance, epidemiology and end results databases (SEER) collected cancer incidence rate data from the population-based cancer registry, covering about 34.6% of the US population. The SEER registry collected data on patient demographics, primary tumor location, tumor morphology, diagnostic stage and the first course of treatment, and tracked the patient's life status. We performed this retrospective analysis based on the SEER 18 registry database (1975–2016 varying).

#### Inclusion criteria

Patients fitting the following criteria were included: primary site labelled at the breast (C50.0 ~ C50.9) according to ICO-3/WHO 2008, diagnosed with definitely pathological confirmation, aged over 18 years old, no distant metastasis, non-inflammatory breast cancer, surgery performed, received preoperative or postoperative RT.

#### Exclusion criteria

Patients fitting the following criteria were excluded: aged less than 18 years old, diagnosis of metastasis breast cancer, unable to identify tumor stage, diagnosis of inflammatory breast cancer, bilateral or unknown lateral breast cancer, no operation or unclear operation method, no or unknown RT.

### Methods

#### Grouping

Selected patients were divided into two groups: those who received localized breast radiation before surgery (preoperative RT) and those who received localized breast radiation after surgery (postoperative RT). Variables included age at diagnosis (≤ 65, > 65), race (white, black, other/ unknown), sex (male, female), marital status at diagnosis (married, unmarried, unknown), grade (I, II, III, IV, unknown), laterality (right, left), histology (ductal, lobular, other), primary site (central, inner, other/unknown), American Joint Committee on Cancer (AJCC) 6th stage (I, II, III), molecular subtypes (Luminal A; Luminal B; HER-2 enriched; Triple negtive), surgery mode (breast-conserving surgery [BCS], mastectomy) and chemotherapy (yes or no/unknown).

#### Statistical analysis

The clinicopathological features were compared by Pearson chi square test. The overall survival (OS) and breast cancer specific survival (CSS) were defined as the time interval from diagnosis to death for any cause or breast cancer. We used stratified log-rank for the primary statistical test and interaction tests for subgroups analysis. Stratification factors included diagnostic age, race, marital status, tumour grade, primary site, molecular subtype, stage, surgical procedure and chemotherapy (Table 1). Kaplan–Meier analysis was performed to compare survival between subgroups. Cox regression model was established to evaluate the independent association with OS and CSS, and the hazard ratio (HR) and its 95% confidence interval (CI) were estimated. The interaction test used for the subgroups was an interaction term added to the Cox regressions. Given the differences between patients receiving preoperative RT and postoperative RT, propensity score matching (PSM) was used to balance measurable confounding factors^15^. We used optimal method with caliper and tolerated maximal distance was 0.05. Patients were matched according to their estimated propensity and replaced with a 1:1 match. Statistical analysis was performed using SPSS 23.0 (Chicago, IL, USA), STATA 17.0 (https://www.stata.com/) and the R programming language (version 4.0.3; https://www.r-project.org/) in RStudio (version 1.3.1093; https://www.rstudio.com/). Two-sided P values < 0.05 were considered statistically significant. In addition, GraphPad Prism9 software was used to draw Kaplan–Meier curves.

**Table 1:** Baseline characteristics of the preoperative and postoperative RT cohorts.

Characteristics	Radiotherapy				P
	Before surgery		After surgery		
	NO	%	NO	%	
**Age**					< 0.001
≤ 65	1224	71.5	270,272	66.0	
> 65	488	28.5	139,295	34.0	
**Sex**					0.105
Female	1702	99.4	408,128	99.6	
Male	10	0.6	1439	0.4	
**Race**					< 0.001
White	1350	78.9	337,328	82.4	
Black	250	14.6	39,071	9.5	
Other/unknown	112	6.5	33,168	8.1	
**Marital status**					< 0.001
Married	924	54.0	243,441	59.4	
Unmarried	699	40.8	152,315	37.2	
Unknown	89	5.2	13,811	3.4	
**Histology**					0.522
Ductal	1288	75.2	307,460	75.1	
Lobular	130	7.6	33,960	8.3	
Other	294	17.2	68,147	16.6	
**Grade**					< 0.001
I	241	14.1	94,813	23.1	
II	592	34.6	169,496	41.4	
III	699	40.8	122,998	30.0	
IV	27	1.6	3496	0.9	
Unknown	153	8.9	18,764	4.6	
**Primary site**					< 0.001
Central	102	6.0	19,119	4.7	
Inner	237	13.8	74,852	18.3	
Other/unknown	1373	80.2	315,596	77.1	
**Laterality**					0.130
Left	834	48.7	207,036	50.5	
Right	878	51.3	202,531	49.5	
**Stage**					< 0.001
I	569	33.2	222,406	54.3	
II	578	33.8	132,153	32.3	
III	565	33.0	55,008	13.4	
**Selected patients were divided into two**					< 0.001
Luminal A	273	15.9	118,079	28.8	
Luminal B	66	3.9	14,194	3.5	
HER-2 enriched	41	2.4	5411	1.3	
Triple negative	103	6.0	15,849	3.9	
Unknown	1229	71.8	256,034	62.5	
**Surgery**					< 0.001
BCS	873	51.0	331,695	81.0	
Mastectomy	839	49.0	77,872	19.0	
**Chemotherapy**					< 0.001
Yes	1130	66.0	183,412	44.8	
No/unknown	582	34.0	226,155	55.2	

The authors declare that all methods were performed in accordance with the relevant guidelines and regulations. Ethical approval was not required because this study does not involve animal or human trials. All data supporting the findings of this study are available within the paper and its supplementary information files.

## Results

### Patient characteristics

We identified 411,279 patients with malignant breast cancer from 18 registries dating from 1988 to 2016 (Supplementary Fig. 1). In this cohort, 1,712 patients were divided into the preoperative RT group, and 409,567 patients were divided into the postoperative RT group. The preoperative and postoperative RT cohorts had different distributions of potentially confounding factors (Table 1).

As shown in Table 1, patients in the preoperative RT group were significantly younger (P < 0.001), more likely to be black (P < 0.001) and unmarried (P < 0.001). Also, compared with the postoperative RT group, the tumours in the preoperative RT group were more aggressive, presenting with more inadequate differentiation, later stage, and higher probability of being TNBC (all P < 0.001). Accordingly, the proportion of patients receiving mastectomy and chemotherapy was higher in the preoperative RT group (all P < 0.001).

### Survival benefit of radiation after surgery over radiation before surgery

**Figure 1:**
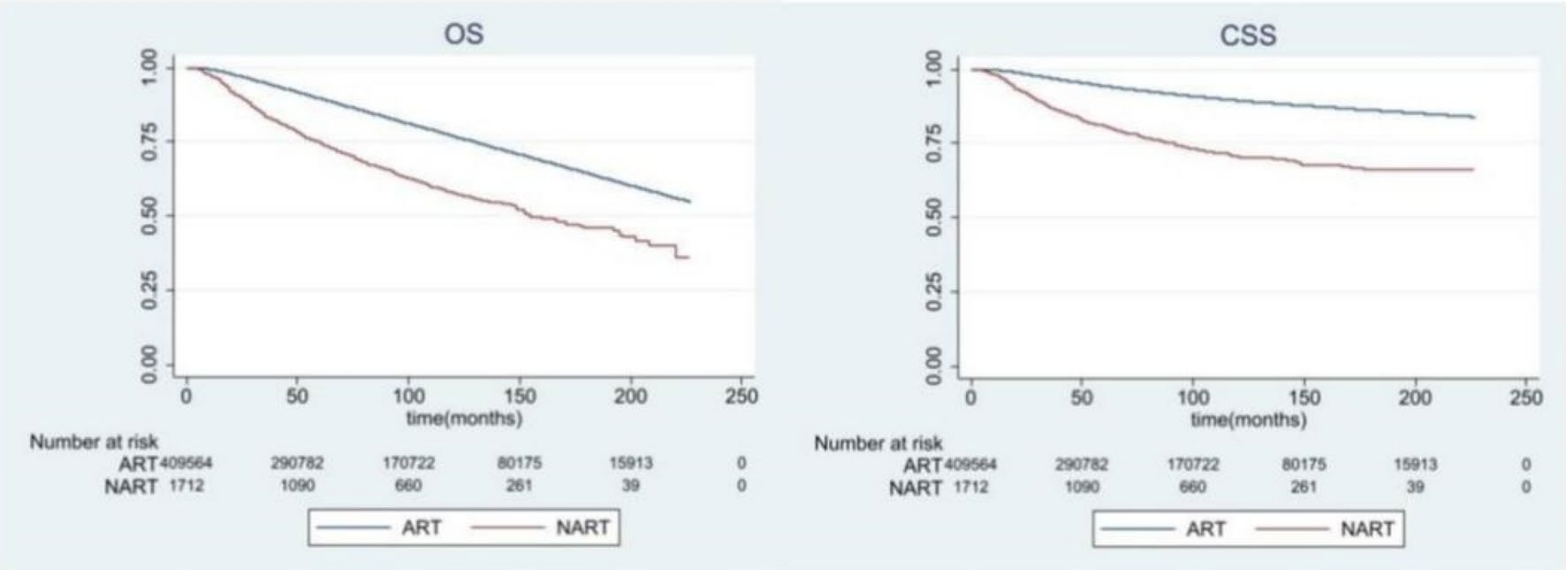
Overall and breast cancer-specific survival by radiation sequence with surgery among the whole cohort.

After median following-up of 84 months, the overall and breast cancer–specific survival was significantly better for the postoperative RT group than the preoperative RT group (both P < 0.0001, Fig. 1). Subgroups included age, sex, race, marital status, tumour grade, primary site, molecular subtype, stage, surgical procedure and chemotherapy. Considering the impact of other variables on survival, we further conducted a subgroup survival analysis. As results, all subgroups without matching showed that postoperative radiation was better than preoperative radiation in the respect of OS and CSS (both P < 0.0001, Supplementary Figs. 2, 3). We then performed Cox analysis (see Tables 2, 3 for results), and the results of single-factor analysis showed patients who received radiation after surgery had better OS (HR  2.111, 95% CI 1.953–2.281, P < 0.001) and CSS (HR 3.215, 95% CI 2.916–3.546, P < 0.001) than their counterparts. After multivariate analysis, we found that RT sequence was still an independent factor affecting OS and CSS. In addition, there was no significant difference in histology, laterality, primary site, and molecular subtype between Luminal A and Luminal B groups in OS; there was no significant difference in age, gender, laterality and primary site in CSS. Median year of diagnosis in preoperative and postoperative group RT was 2006 and 2008 respectively.

**Table 2:** Univariate and multivariate Cox regression of overall survival in the whole cohort.

Characteristics	Univariate			Multivariate		
	HR	95% CI	P	HR	95% CI	P
**Ages (years)**						
≤ 65	Reference			Reference		
> 65	2.861	2.822–2.900	< 0.001	3.002	2.957–3.048	< 0.001
**Sex**						
Female	Reference			Reference		
Male	2.445	2.246–2.661	< 0.001	1.276	1.171–1.391	< 0.001
**Race**						
White	Reference			Reference		
Black	1.368	1.339–1.398	< 0.001	1.259	1.232–1.288	< 0.001
Other/unknown	0.724	0.703–0.746	< 0.001	0.805	0.782–0.830	< 0.001
**Marital status**						
Married	Reference			Reference		
Unmarried	1.698	1.675–1.722	< 0.001	1.386	1.366–1.406	< 0.001
Unknown	1.328	1.275–1.382	< 0.001	1.221	1.172–1.271	< 0.001
**Histology**						
Ductal	Reference			Reference		
Lobular	1.191	1.163–1.220	< 0.001	0.982	0.957–1.007	0.163
Other	0.981	0.963–0.999	0.035	0.956	0.938–0.973	< 0.001
**Grade**						
I	Reference			Reference		
II	1.291	1.265–1.316	< 0.001	1.178	1.155–1.202	< 0.001
III	1.773	1.738–1.808	< 0.001	1.597	1.563–1.632	< 0.001
IV	1.643	1.547–1.745	< 0.001	1.532	1.442–1.628	< 0.001
Unknown	1.450	1.403–1.499	< 0.001	1.244	1.203–1.287	< 0.001
**Laterality**						
Left	Reference			Reference		
Right	–	–	0.189	–	–	–
**Primary site**						
Central	Reference			Reference		
Inner	0.730	0.707–0.754	< 0.001	0.973	0.942–1.006	0.106
Other/unknown	0.789	0.767–0.812	< 0.001	0.947	0.920–0.975	< 0.001
**Stage**						
I	Reference			Reference		
II	1.395	1.373–1.417	< 0.001	1.524	1.497–1.552	< 0.001
III	2.957	2.906–3.008	< 0.001	2.967	2.895–3.041	< 0.001
**Subtype (2010 +)**						
Luminal A	Reference			Reference		
Luminal B	1.027	0.955–1.105	0.471	0.982	0.913–1.057	0.633
HER-2 enriched	1.677	1.530–1.837	< 0.001	1.415	1.291–1.551	< 0.001
Triple negative	2.808	2.680–2.941	< 0.001	2.476	2.361–2.596	< 0.001
Unknown	1.358	1.323–1.395	< 0.001	1.365	1.329–1.402	< 0.001
**Surgery mode**						
BCS	Reference			Reference		
Mastectomy	2.100	2.069–2.132	< 0.001	1.429	1.401–1.458	< 0.001
**Chemotherapy**						
Yes	Reference			Reference		
No/unknown	1.042	1.028–1.056	< 0.001	1.356	1.331–1.381	< 0.001
**Radiation**						
After surgery	Reference			Reference		
Before surgery	2.111	1.953–2.281	< 0.001	1.671	1.546–1.806	< 0.001

**Table 3:** Univariate and multivariate Cox regression of breast cancer-specific survival in the whole cohort.

Characteristics	Univariate			Multivariate		
	HR	95% CI	P	HR	95% CI	P
**Ages (years)**						
≤ 65	Reference			Reference		
> 65	–	–	0.359	–	–	–
**Sex**						
Female	Reference			Reference		
Male	2.294	2.009–2.620	< 0.001	–	–	0.172
**Race**						
White	Reference			Reference		
Black	1.837	1.782–1.893	< 0.001	1.296	1.256–1.337	< 0.001
Other/unknown	0.922	0.883–0.962	< 0.001	0.844	0.809–0.881	< 0.001
**Marital status**						
Married	Reference			Reference		
Unmarried	1.313	1.285–1.342	< 0.001	1.248	1.220–1.276	< 0.001
Unknown	1.148	1.078–1.222	< 0.001	1.144	1.074–1.218	< 0.001
**Histology**						
Ductal	Reference			Reference		
Lobular	1.178	1.135–1.222	< 0.001	1.141	1.097–1.188	< 0.001
Other	0.849	0.824–0.875	< 0.001	0.948	0.920–0.977	0.001
**Grade**						
I	Reference			Reference		
II	2.783	2.659–2.912	< 0.001	1.914	1.828–2.004	< 0.001
III	6.542	6.262–6.835	< 0.001	3.230	3.084–3.383	< 0.001
IV	6.422	5.888–7.004	< 0.001	3.286	3.010–3.588	< 0.001
Unknown	3.492	3.280–3.717	< 0.001	2.109	1.978–2.249	< 0.001
**Laterality**						
Left	Reference			Reference		
Right	0.976	0.955–0.997	0.023	–	–	0.173
**Primary site**						
Central	Reference			Reference		
Inner	0.688	0.653–0.724	< 0.001	1.067	1.013–1.124	0.015
Other/unknown	0.818	0.781–0.856	< 0.001	0.960	0.917–1.005	0.081
**Stage**						
I	Reference			Reference		
II	3.454	3.352–3.559	< 0.001	2.575	2.491–2.663	< 0.001
III	10.872	10.560–11.193	< 0.001	6.152	5.918–6.396	< 0.001
**Subtype (2010 +)**						
Luminal A	Reference			Reference		
Luminal B	1.288	1.163–1.426	< 0.001	0.767	0.693–0.850	< 0.001
HER-2 enriched	2.703	2.413–3.028	< 0.001	1.265	1.128–1.418	< 0.001
Triple negative	4.934	4.649–5.235	< 0.001	2.780	2.616–2.954	< 0.001
Unknown	1.743	1.675–1.815	< 0.001	1.478	1.419–1.539	< 0.001
**Surgery mode**						
BCS	Reference			Reference		
Mastectomy	4.490	4.395–4.587	< 0.001	1.614	1.571–1.658	< 0.001
**Chemotherapy**						
Yes	Reference			Reference		
No/unknown	0.319	0.312–0.327	< 0.001	1.080	1.049–1.111	< 0.001
**Radiation**						
After surgery	Reference			Reference		
Before surgery	3.215	2.916–3.546	< 0.001	1.834	1.663–2.023	< 0.001

**Figure 2:**
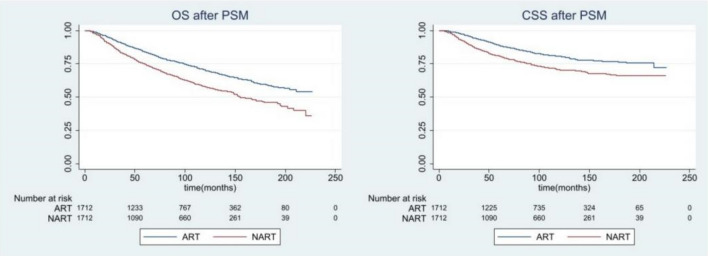
Overall and breast cancer-specific survival by radiation sequence with surgery among the whole cohort after PSM.

According to the results of Cox univariate and multivariate analysis, we further conducted PSM on the general population and balanced the other significant variables. The median following-up time was 82.5 months. As seen from the Fig. 2, OS and CSS adjusted by PSM were higher for patients who underwent postoperative RT than those who received preoperative RT. Besides, the relationship between the breast cancer specific death and other causes of death showed in the Supplemetary Material 6. Although the analysis of subgroups after stratification still showed that the survival curve after the operation was better than that before the operation, it can be seen from the survival curve that the two lines of some subgroups in staging and molecular typing overlapped. Therefore, we carried out the subgroup analysis of staging and molecular typing in the next step.

### Survival benefit analysis between preoperative and postoperative RT by stage

**Figure 3:**
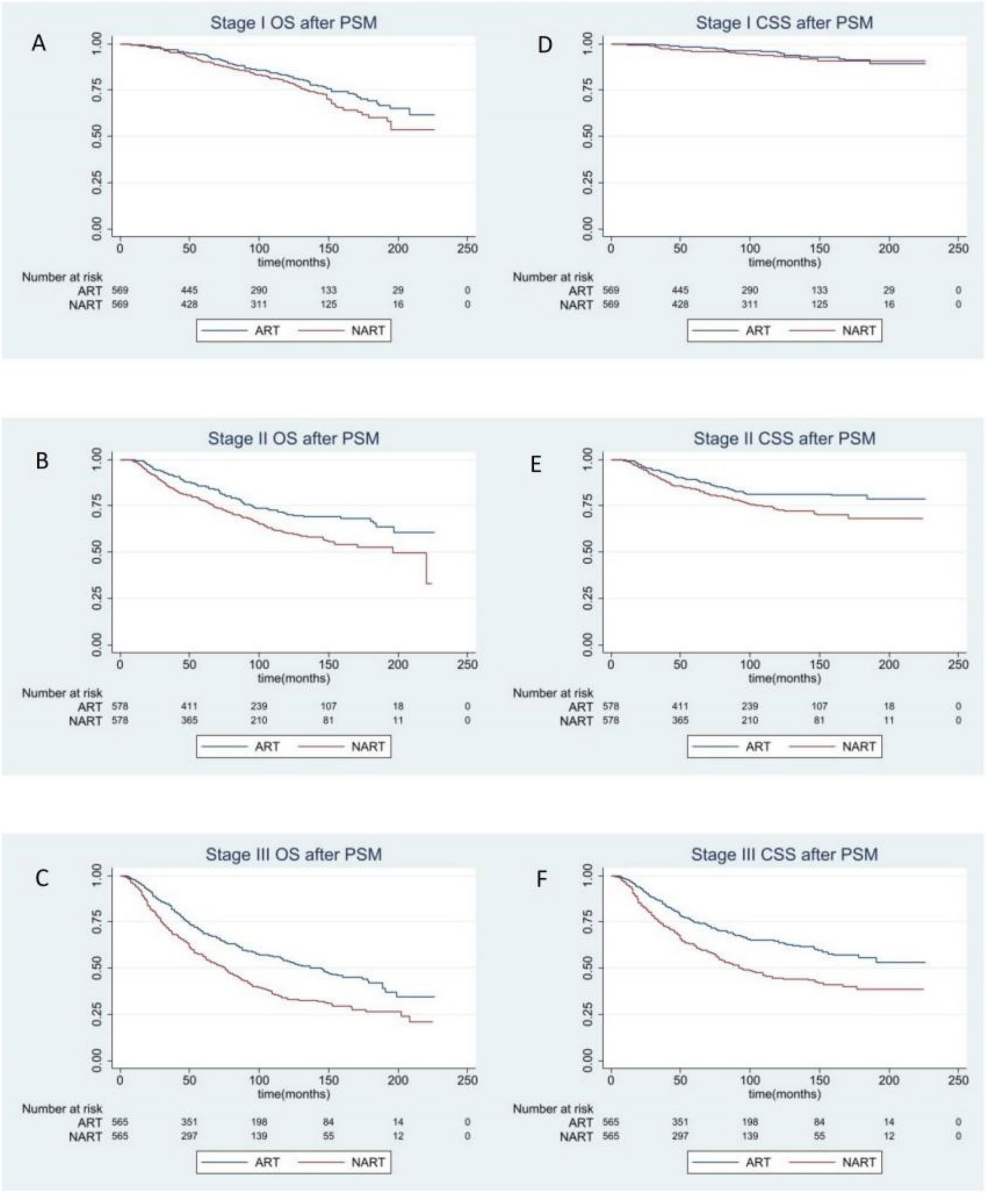
Overall and breast cancer–specific survival by radiation sequence with surgery in the stage subgroup based on propensity score (PS) matching-adjusted survival data. All *P* values based on PS matching-adjusted, two-sided log-rank test. Left column, overall survival: (**A**) stage I, (**B**) stage II, (**C**) stage III. Right column, breast cancer–specific survival: (**D**) stage I, (**E**) stage II, (**F**) stage III.

Because tumor staging is an important prognostic factor, we further stratify patients according to AJCC staging. AJCC staging is one of the main indicators of breast cancer oriented surgery in NCCN guidelines. We analyzed the OS and CSS of two groups of patients. After median following-up of 105 months, as results, a significant correlation between PSM adjusted OS and CSS and AJCC staging was observed (Fig. 3). Specifically, there was no significant difference in OS (P = 0.0545) or CSS (P = 0.0957) between the postoperative RT and preoperative RT groups among stage I. By comparison, patients of stage II and III exhibited marked increases in OS (stage II: P ≤ 0.0001; stage III: P < 0.0001) and CSS (stage II: P = 0.0025; stage III: P < 0.0001) in postoperative RT group than preoperative RT group (Supplementary Fig. 4).

### Survival benefit analysis between preoperative RT and postoperative RT by molecular subtype

**Figure 4:**
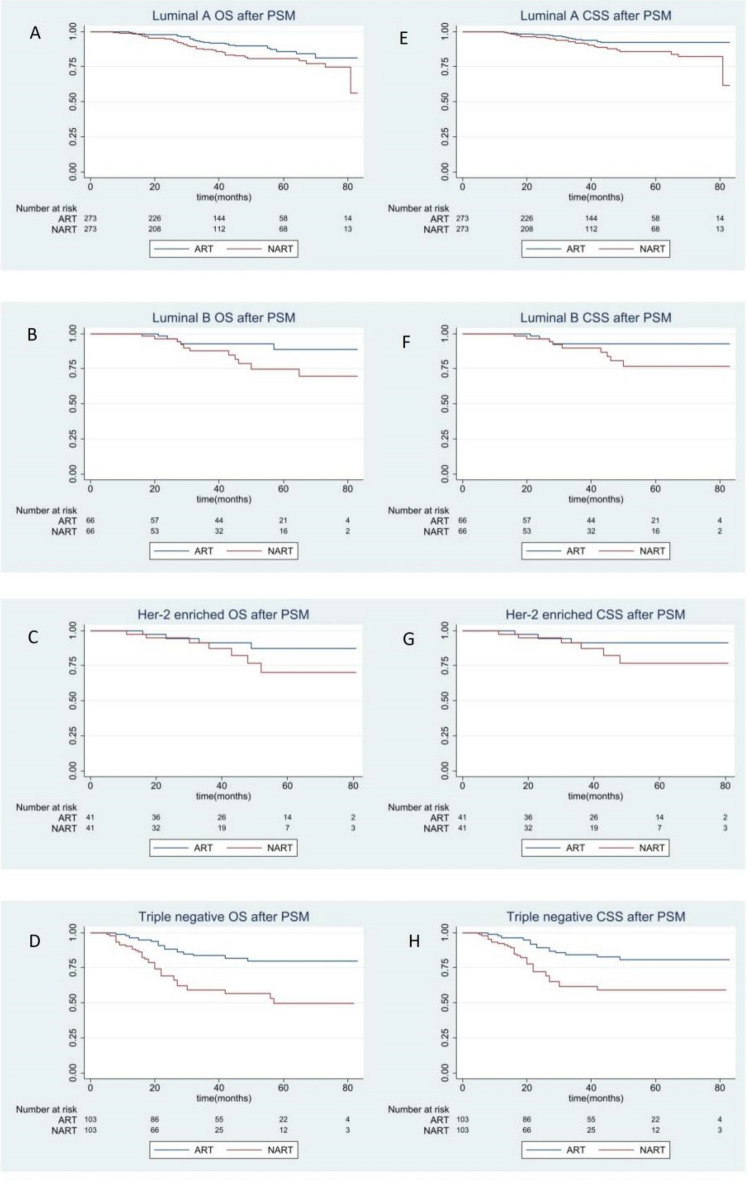
Overall and breast cancer–specific survival by radiation sequence with surgery in the molecular subtype subgroup based on propensity score (PS) matching-adjusted survival data. All *P* values based on PS matching-adjusted, two-sided log-rank test. Left column, overall survival: (**A**) Luminal A, (**B**) Luminal B, (**C**) HER-2 enriched, (**D**) Triple negative. Right column, breast cancer-specific survival: (**E**) Luminal A, (**F**) Luminal B, (**G**) HER-2 enriched, (**H**) Triple negative.

In breast cancer, the subtype is also important for treatment, survival and prognosis. After matching, other variables between the two groups were balanced. The median following-up time was 37 months. Among molecular subtype subgroup, patients with TNBC obtained overall survival benefit from postoperative RT (P < 0.0001, Fig. 4D). Similarly, good CSS was observed in postoperative RT patients (P = 0.0006, Fig. 4H). Yet, no OS or CSS difference between two groups was observed in Luminal A, Luminal B, Her-2 enriched cohorts (OS: P = 0.0316, P = 0.0619, P = 0.2061, respectively; CSS: P = 0.0201, P = 0.0859, P = 0.2140, respectively; Fig. 4A–C,E,F,G; Supplementary Fig. 5).

## Discussion

RT plays a significant role in the comprehensive treatment of breast cancer. The role of RT is mainly reflected in the following aspects. First, RT as a radical treatment is combined with breast-conserving surgery to achieve the same effect as total mastectomy. Secondly, the local control rate and overall survival rate of patients with high recurrence risk were significantly improved. Last but not least, RT is the primary means of palliative treatment for metastatic breast cancer because it can effectively relieve symptoms, relieve pain and improve the quality of life of patients. Although postoperative RT significantly improved the prognosis of patients with early breast cancer after breast conserving surgery, only 63% of patients had no cancer in 20 years of initial treatment^2^.

RT can enhance tumor specific immune response in established tumors^16–22^. Recent studies have shown that radiation from large tumors can activate a strong anti-tumor immune response^23,24^, and it is possible to transform tumors into patient specific in situ vaccines that can re-educate the immune system to recognize and reject cancer^25,26^. Therefore, it is conceivable that preoperative RT applied to most diseases will activate strong anti-tumor immunity, which does not exist after postoperative RT on the tumor bed. Radiation induced anti-tumor immunity may help to eradicate subclinical diseases and distant micrometastasis in ipsilateral and contralateral breasts, which may lead to immune memory, so as to vaccinate future tumors^25^. This hypothesis inspired the analysis of the long-term results of preoperative RT and standard care postoperative RT in cancer patients.

Our data confirmed the critical role of radiation in the multidisciplinary management of breast cancer^5,27^. Patients receiving preoperative RT had a significantly higher risk of worse OS and CSS than patients receiving postoperative RT. We analyze the reasons that preoperative RT is not better than postoperative RT, mainly in the following aspects.

Primarily, the time of conventional preoperative RT is too long, which may affect the survival. At present, the RT technology has been updated. The time of RT technology such as accelerated partial breast irradiation (APBI) and hypofractionation is shorter, allowing more accurate location for tumor and having little affect to other treatments.

There are only a few studies evaluating the efficacy and effectiveness of preoperative PBI. One of the benefits of preoperative PBI is that it improves the visibility of the primary tumor, makes the target area smaller and more accurate, and minimizes the risk of geographic error. In addition, surgery is performed after preoperative PBI, so the breast area receiving the highest radiation dose can be removed, which may lead to limited fibrosis and good cosmetic results^28^. In general, the published series of reports have gained experience from a small number of cases and a short median follow-up time (range 16.2–43.2 months)^28–32^. Preoperative stereotactic whole body RT (SBRT) and radiosurgery (SRS) may be also a new potential method of breast cancer multimodal treatment. SRS/SBRT allows each part of a higher dose to be provided in one or several parts, which can improve patient compliance and consume fewer medical resources. Bondiau et al. conducted a single-institution phase I study to find the maximum tolerated dose of SBRT system in 3 fraction preoperatively administered to locally advanced breast cancer patients receiving neoadjuvant chemotherapy^30^. Nine out of 25 patients (36%) had a complete pathological response (pCR). At dose of 25.5 Gy, the pCR rate reached 67%, and at dose levels of 28.5 Gy and 31.5 Gy, the pCR rate reached 43% and 33%, respectively. Two patients had non-dose limiting grade 2 toxicity, and one grade 3 dermatological dose limiting toxicity was reported as grade 4.

Secondly, in the survival analysis, a strong association between various types of mortality and clinical stage was observed. In patients diagnosed with stage III cancer, the results of preoperative RT were significantly worse than postoperative RT. Patients with stage I-II cancer may lead to non-significant differences in the treatment of these patients. These results suggest that it is necessary to further evaluate the long-term therapeutic effects of different radiation methods and surgery. Less invasive, more restrained and personalized local treatment strategies based on local recurrence probability and death risk should be considered. Nowadays, neoadjuvant chemotherapy has been applied in breast cancer very well, and its curative effect is clear. However, the chemotherapy part in the database has no further distinction between non-chemotherapy and unknown. There is no subdivision whether neoadjuvant or adjuvant chemotherapy were performed for patients undergoing chemotherapy, which is likely to affect the results of the study.Anthracycline plus taxane-based chemotherapy is the most widely used neoadjuvant chemotherapy (NAC) regimen for all early breast cancer subtypes, and is associated with a high clinical response rate^33^. There was very little progress during NAC. In a meta-analysis of 1928 patients, the progress rate was 3%^34^. In patients with HER-2 positive breast cancer, trastuzumab with or without pertuzumab should be taken at the same time as taxane^1,35,36^. For patients with TNBC, the addition of carboplatin to the GeparSixto^37^ and CALGB 40603^38^ studies showed an increase in pCR rate, despite the increased toxicity, the BCS rate did not increase significantly.

Thirdly, at present, the molecular subtypes of breast cancer are not recommended to guide RT indications; however, many literatures focus on the prognosis of breast cancer subtypes receiving RT in different clinical settings^39–45^. Our study found that TNBC subtype breast cancer can obtain significant overall survival benefit from postoperative RT. Luminal A and HER-2 positive patients (including Luminal B and HER-2 enriched subtypes) are on the verge. The internal mechanism of Luminal A breast cancer radiosensitivity has been proved to be related to ER signaling pathway^46^, epidermal growth factor receptor and downstream signal^47,48^. The cause of radioresistance of HER-2 positive breast tumors is related to the circular HER-2/NF-kB/HER-2 pathway^49^ and epithelial mesenchymal transformation^50^. Therefore, the RT sequence of luminal A and HER-2 positive patients is worthy of further exploration and verification. In view of the lack of treatment for TNBC patients, RT is still an indispensable choice, although the survival rate is limited.

Last but not least, the follow-up time is relatively short, because the HER-2 status in the SEER database is only available in 2010. Therefore, in order to balance the study cohort and follow-up time, we limited the study patients to those diagnosed between 2010 and 2014. So far, neoadjuvant endocrine therapy has been used less frequently than chemotherapy. Aromatase inhibitors are used in selected patient subgroups such as women with larger, hormone-receptor-rich breast cancer after menopause, usually because of tumor biology or patient characteristics that do not require systemic chemotherapy^35,36,51^. This may include patients with positive or negative lymph nodes^51,52^. A trial of 182 patients who received neoadjuvant letrozole treatment showed that the incidence of BCS was 69.8% at 3 months, which rose to 83.5% after 2 years of treatment^53^. A recent meta-analysis of 20 studies showed that neoadjuvant endocrine therapy may be as effective as NAC, but with lower toxicity^54^. Therefore, neoadjuvant endocrine therapy should be considered in selected patients.

This study has certain limitations. First, we can not obtain data on the rate or pattern of recurrence or metastasis leading to the incapacity of analyzing the rate of local recurrence. In addition, there are no other factors with specific guiding indications in the SEER database, such as lymphatic vascular infiltration, extranodal tumor expansion, surgical margin status, irradiation range, and molecular drug management^55^. Besides, the management of chemotherapy regimens and hormone therapy is beyond our reach. Eventually, as a retrospective study, our research may have selection bias.

## Conclusions

In this ever-evolving situation, predicting the possibility of RT before surgery is considered an interesting goal, as confirmed by some of the experiences reported in this article. In the current literature review, available clinical data on the potential impact of preoperative RT on breast cancer treatment is analyzed. The hypothetical role of this preoperative method in the breast cancer setting may be related to two main reasons. One reason is, compared with the postoperative bed, in order to accurately identify local tumor expansion from a targeting perspective. Another is to obtain better results after neoadjuvant treatment. Perhaps, this final treatment goal can prove the clinician's ambition to explore the role of preoperative RT in breast cancer. Compared with standard neoadjuvant system treatment, the pCR rate may be higher.

In conclusion, according to the results of this study, preoperative RT did not show a survival advantage. postoperative RT is still the primary treatment. However, preoperative RT also has some theoretical advantages, such as phase reduction and recurrence reduction. At present, there are no clinical trials in this area, only some retrospective studies, but it is still worthy of further exploration.

## Supplementary Information

Supplementary Figure 1.

Supplementary Figure 2.

Supplementary Figure 3.

Supplementary Figure 4.

Supplementary Figure 5.

Supplementary Information.

## Data Availability

The authors declare that all data supporting the findings of this study are available within the paper and its supplementary information files.
