# Supplementary material for: Survival comparison between postoperative and preoperative radiotherapy for stage I–III non-inflammatory breast cancer
Source: Sci Rep. 2022 Aug 22;12:14288. doi: 10.1038/s41598-022-18251-3 (PMC9395522; doi:10.1038/s41598-022-18251-3)
Supplement: Supplementary file 1 — Supplementary Figure 1. [file 41598_2022_18251_MOESM1_ESM.pdf]

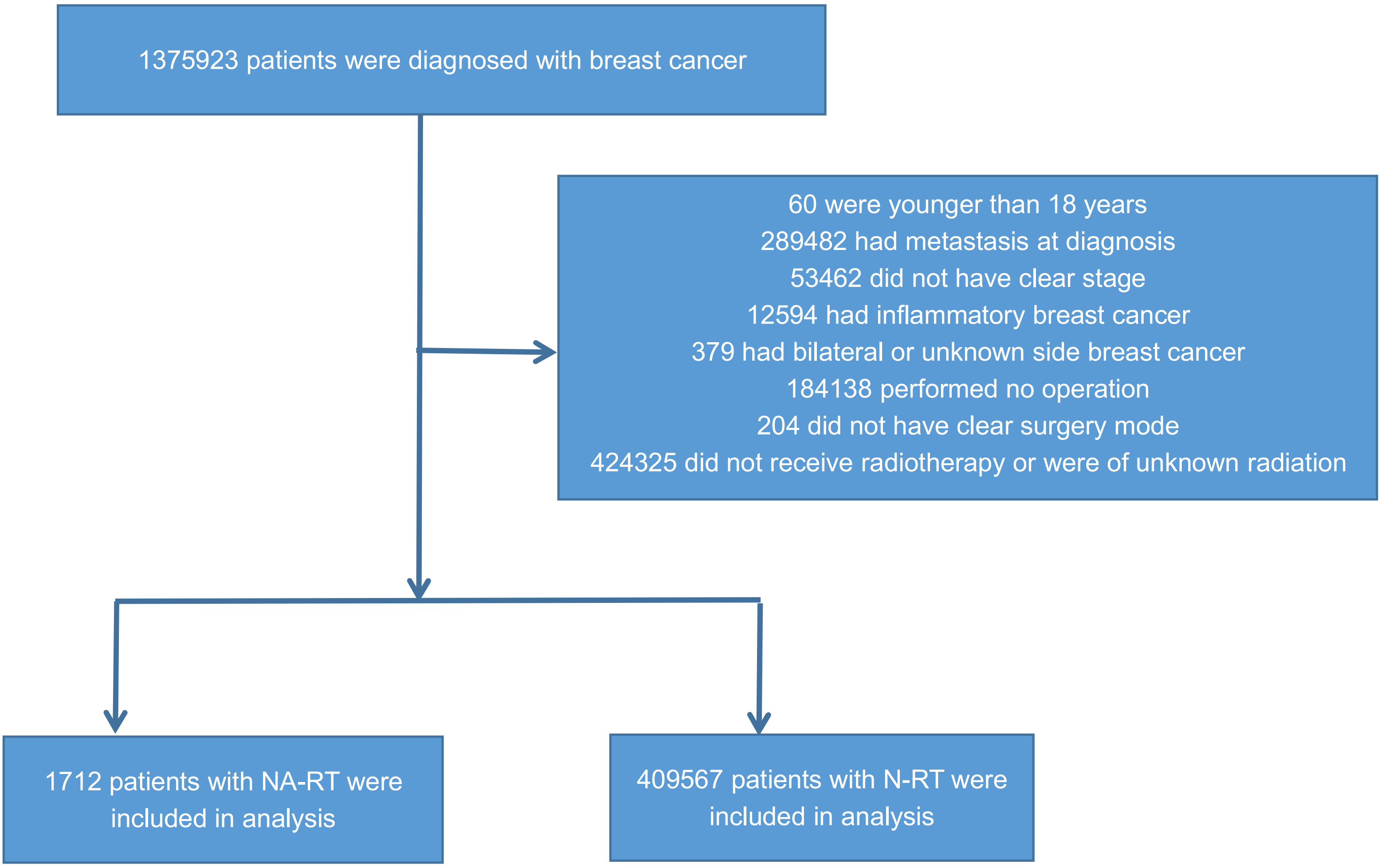


Supplementary Figure.1 Study enrollment of 1375923 breast cancer records present in the Surveillance, Epidemiology, and End Results database, 1712 patients who had radiotherapy (RT) before surgery (NA-RT) and 409567 patients who had RT after surgery (A-RT) were included in the analysis.
