# Supplementary material for: Survival comparison between postoperative and preoperative radiotherapy for stage I–III non-inflammatory breast cancer
Source: Sci Rep. 2022 Aug 22;12:14288. doi: 10.1038/s41598-022-18251-3 (PMC9395522; doi:10.1038/s41598-022-18251-3)
Supplement: Supplementary file 3 — Supplementary Figure 3. [file 41598_2022_18251_MOESM3_ESM.pdf]

Stratum: Age, 1: ≤65, 2: >65

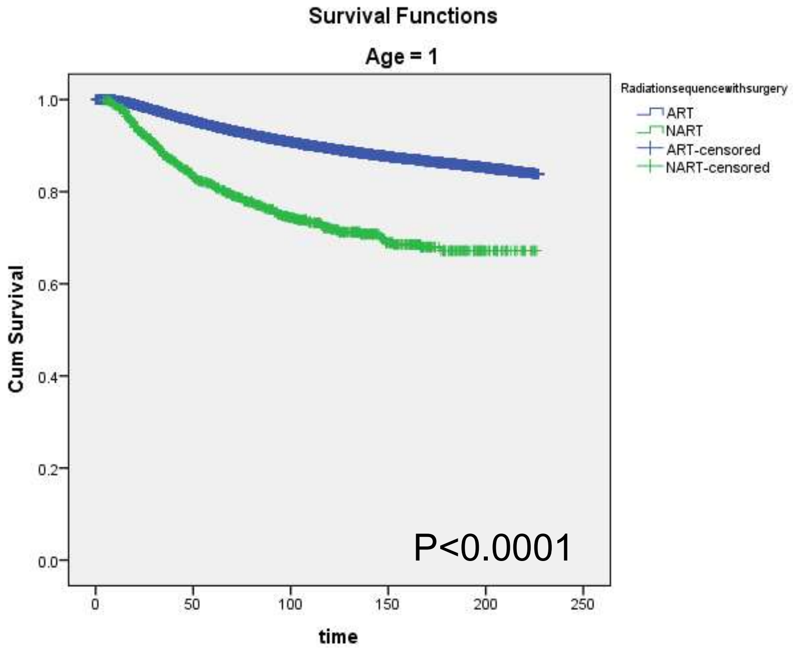

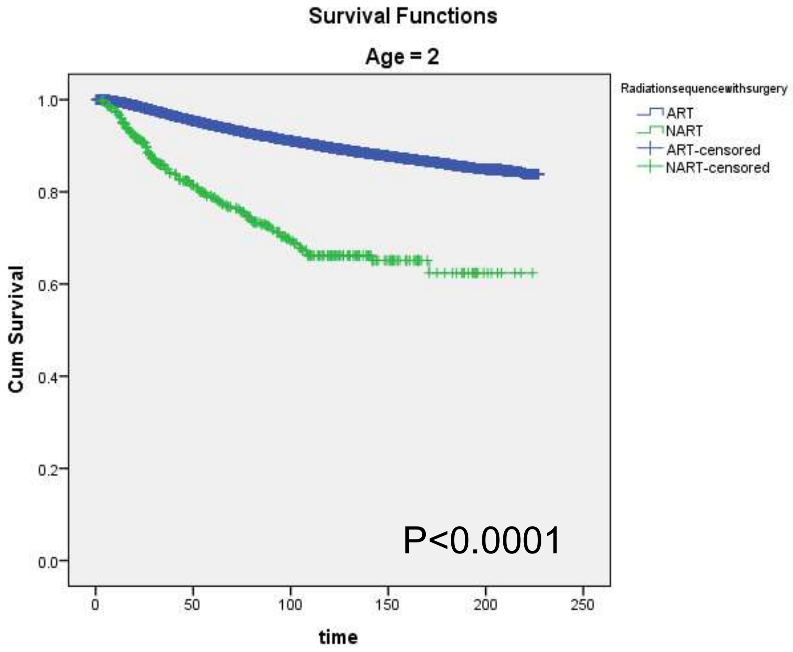

Stratum: Race, 1: white, 2: black, 3: other/unknown

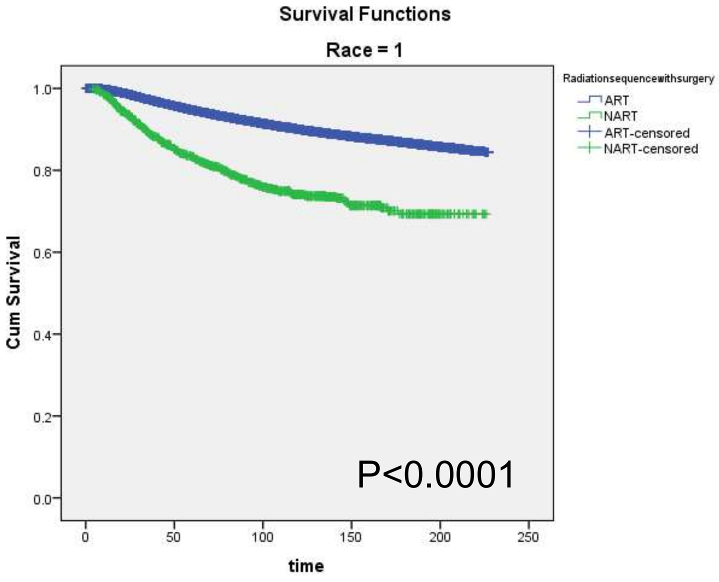

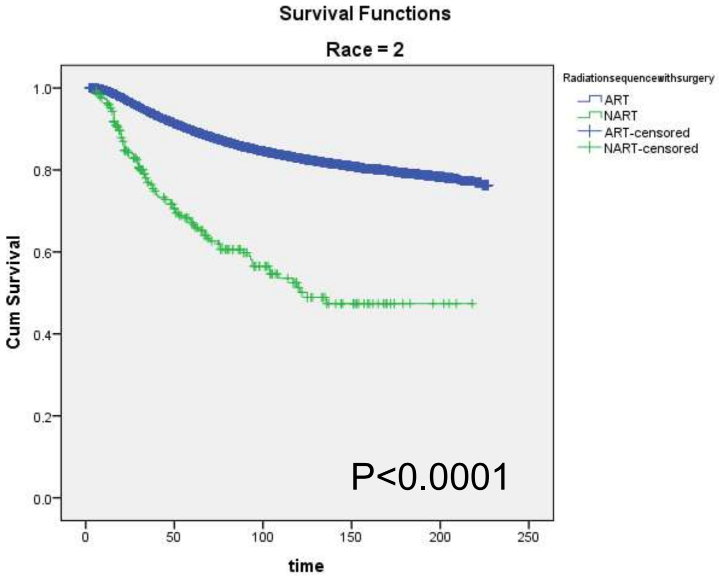

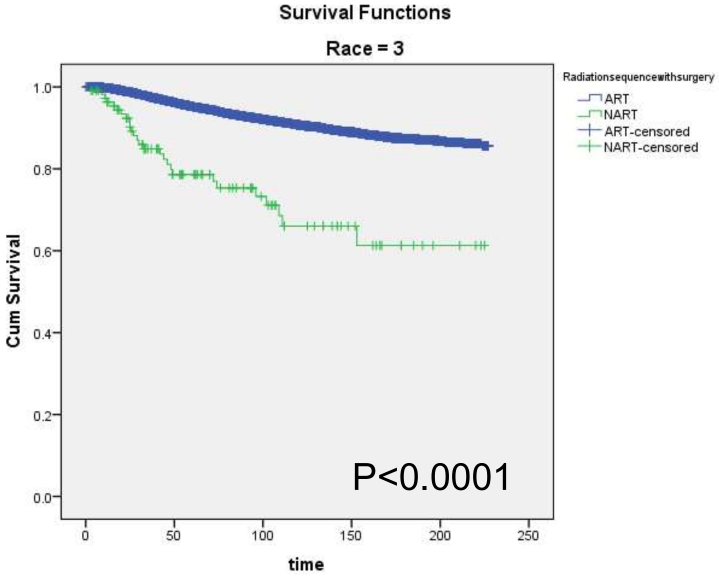

Stratum: Sex, 1: female, 2: male

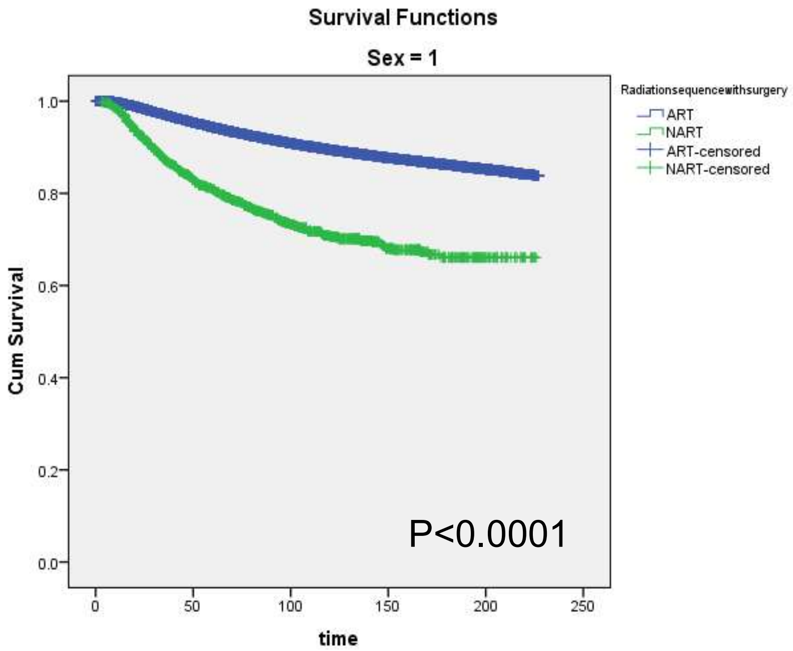

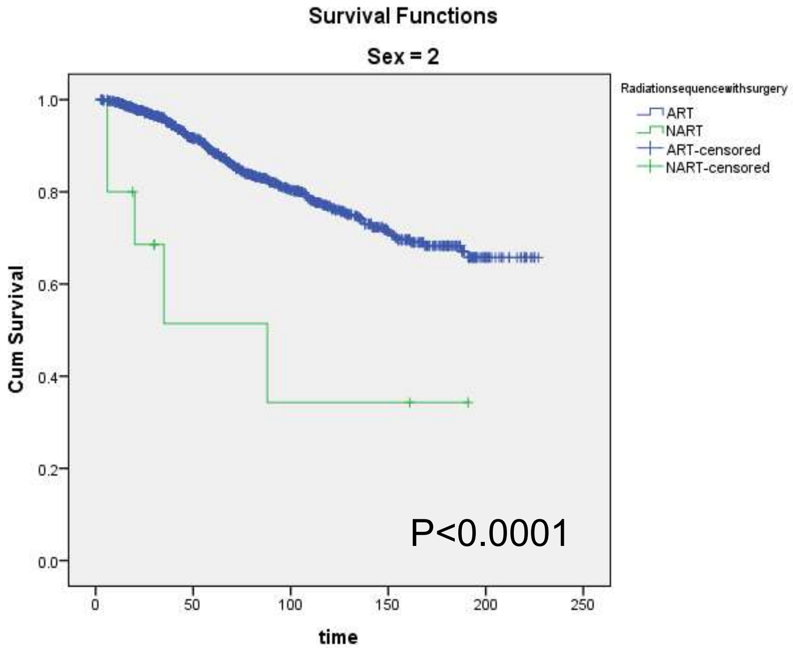

Stratum: Marital status,1: married, 2: unmarried, 3:unknown

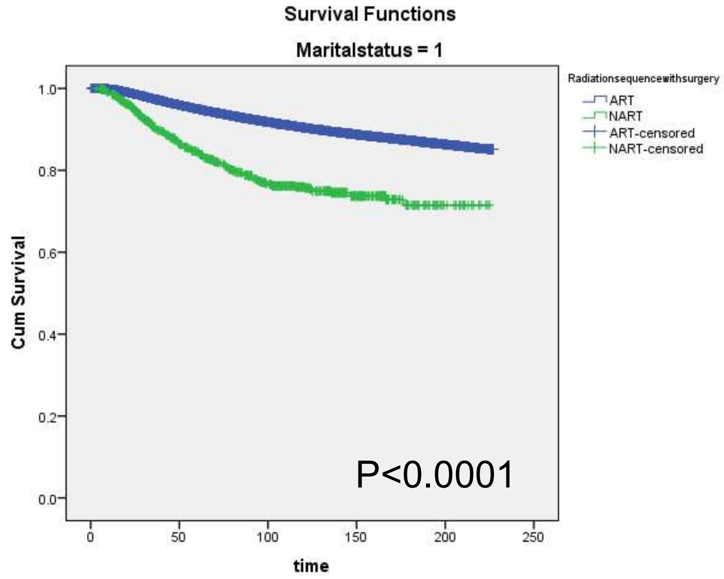

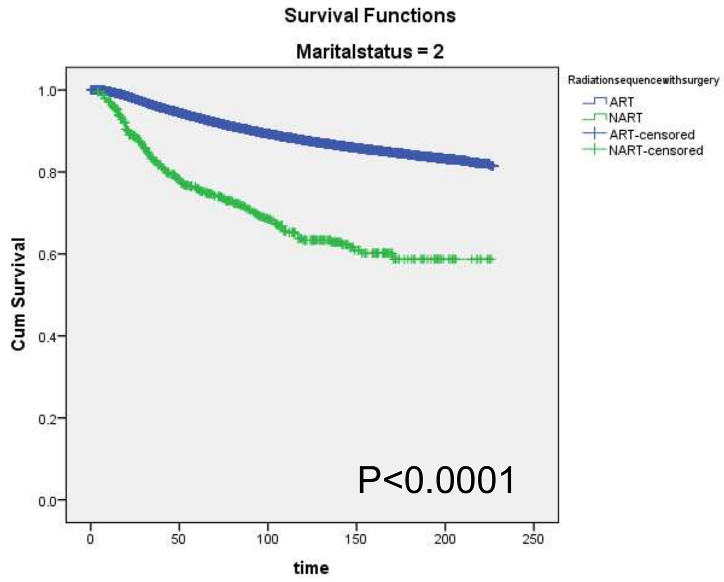

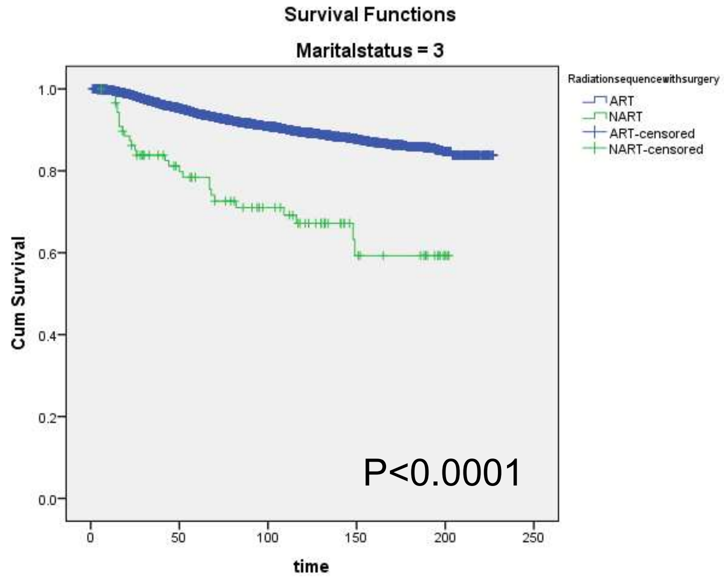

Stratum: Grade, 1: I, 2: II, 3: III, 4: IV, 5: unknown

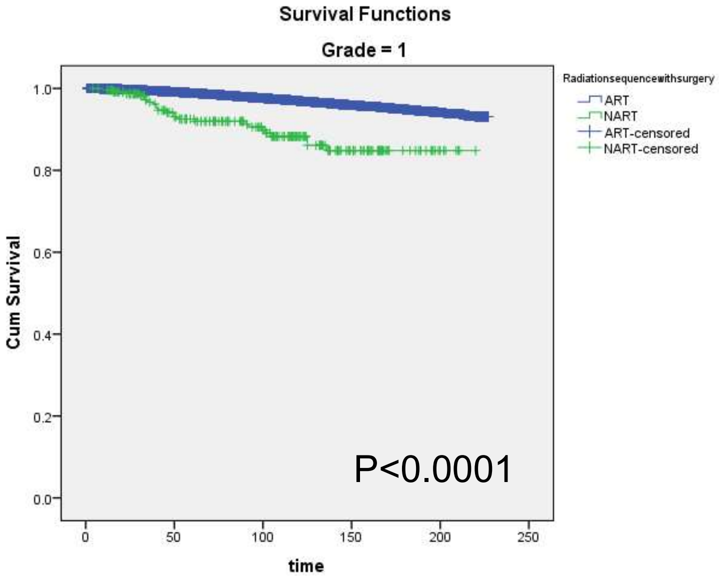

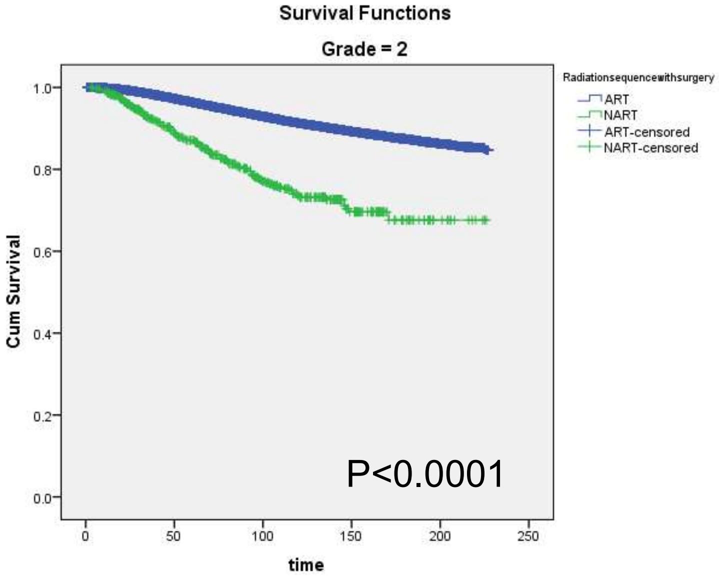

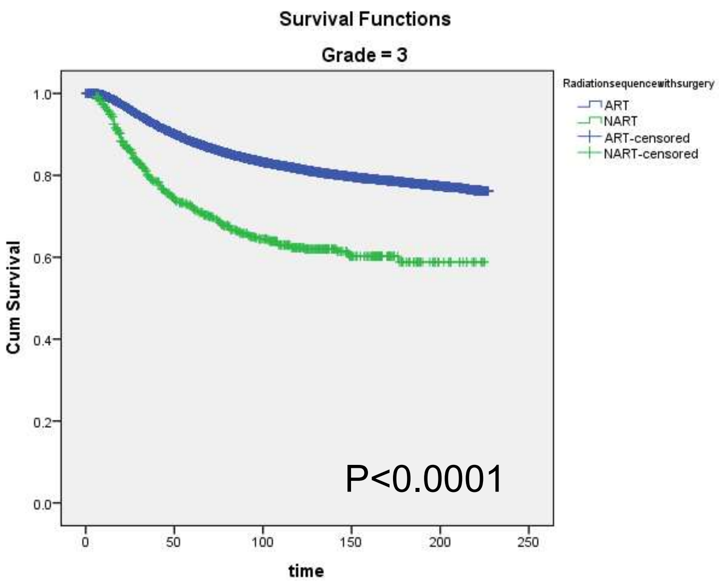
Survival Functions
Grade = 3
Radiationsequencewithsurgery
ART
NART
ART-censored
NART-censored
Cum Survival
1.0
0.8
0.6
0.4
0.2
0.0
0
50
100
150
200
250
time
P<0.0001

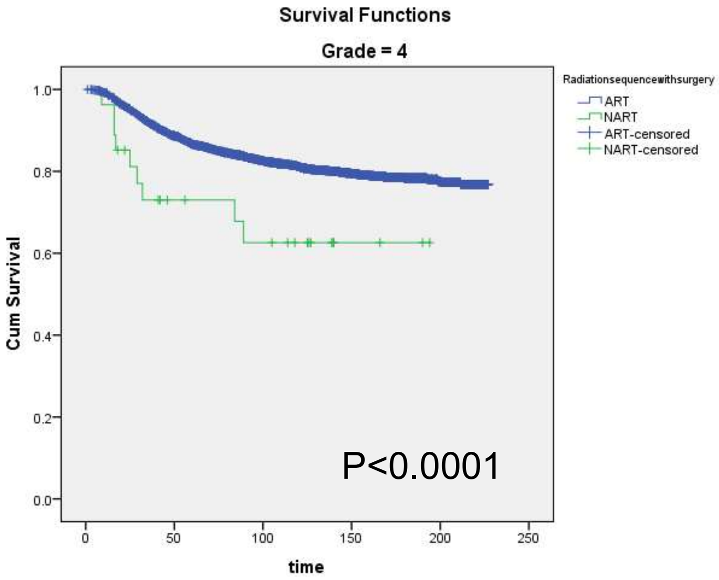
Survival Functions
Grade = 4
Radiationsequencewithsurgery
ART
NART
ART-censored
NART-censored
Cum Survival
1.0
0.8
0.6
0.4
0.2
0.0
0
50
100
150
200
250
time
P<0.0001

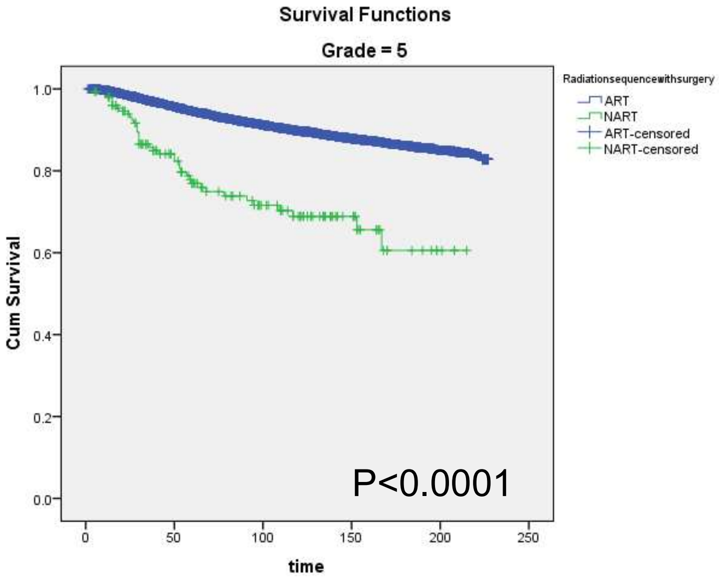
Survival Functions
Grade = 5
Radiationsequencewithsurgery
ART
NART
ART-censored
NART-censored
Cum Survival
1.0
0.8
0.6
0.4
0.2
0.0
0
50
100
150
200
250
time
P<0.0001

Stratum: Histology= 1, 1: ductal, 2: lobular, 3: other

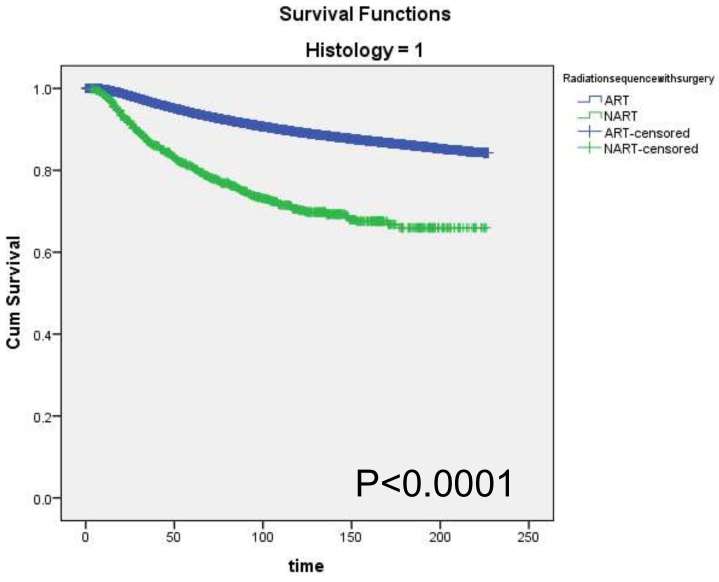

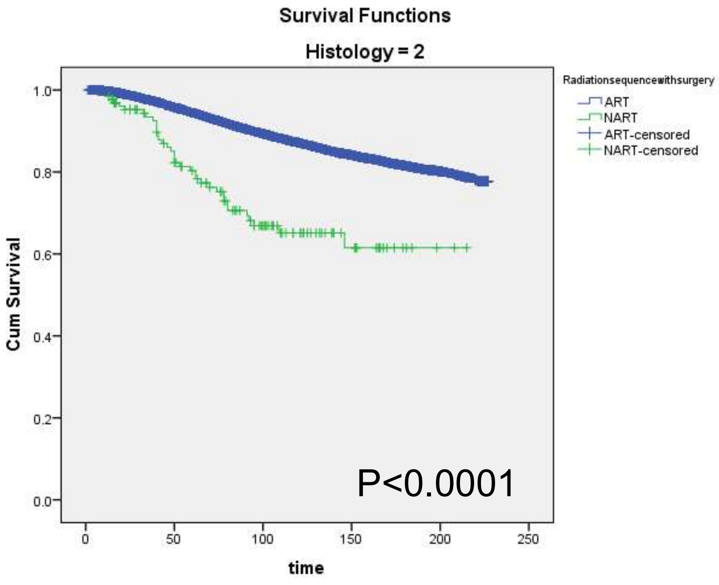

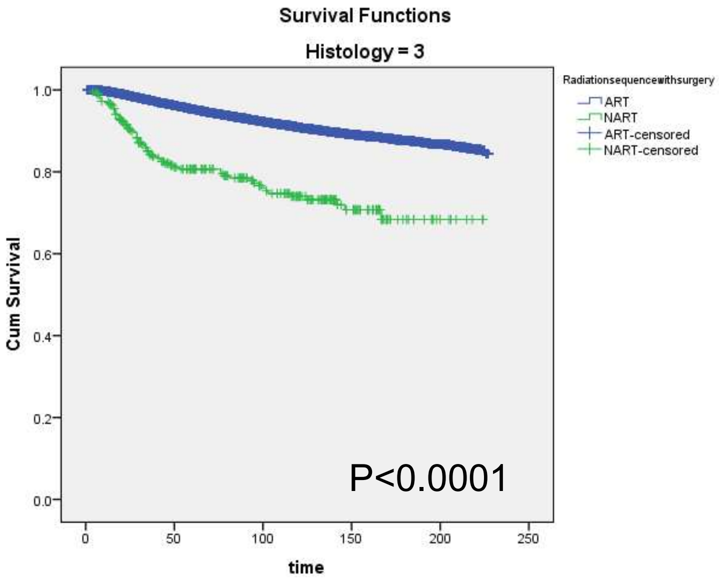

Stratum: Laterality, 1: left, 2: right

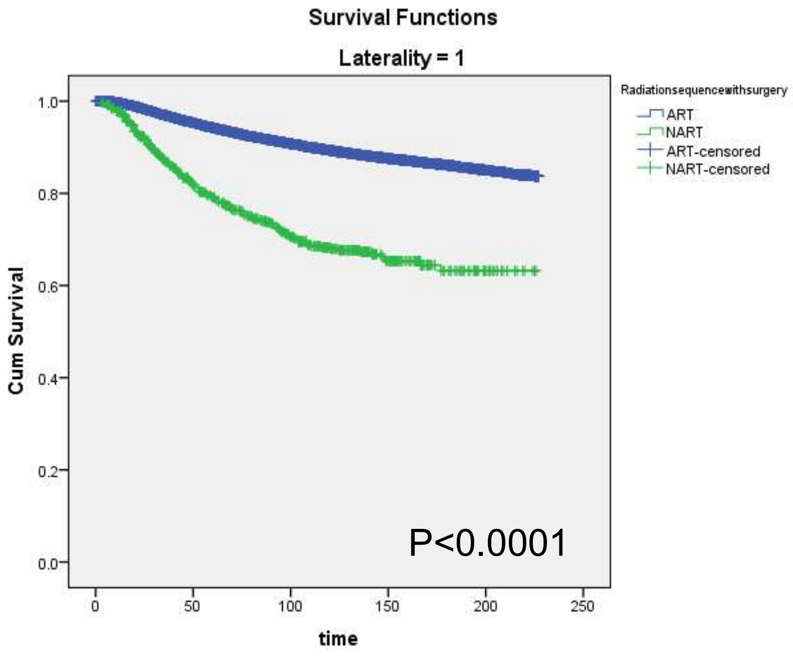

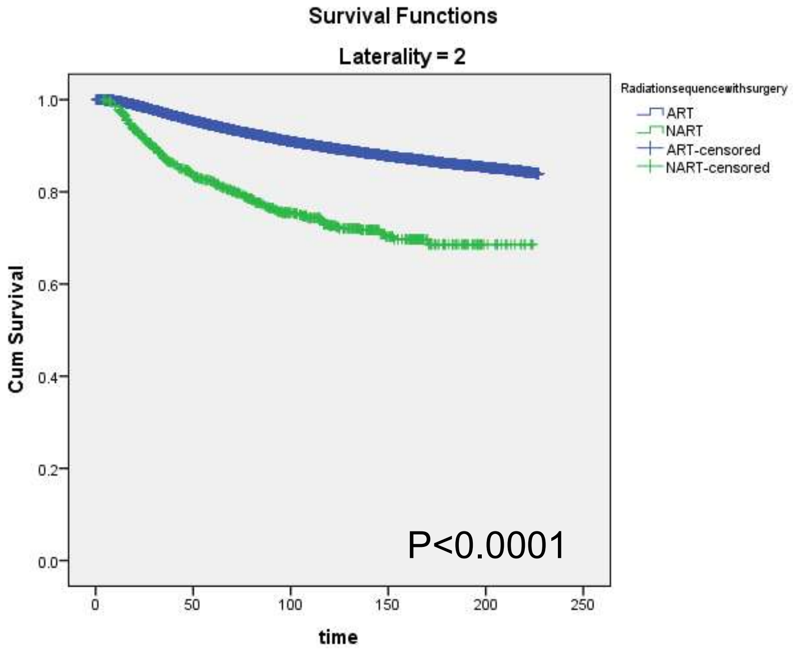

Stratum: Primary site, 1: central, 2: inner, 3: other/unknown

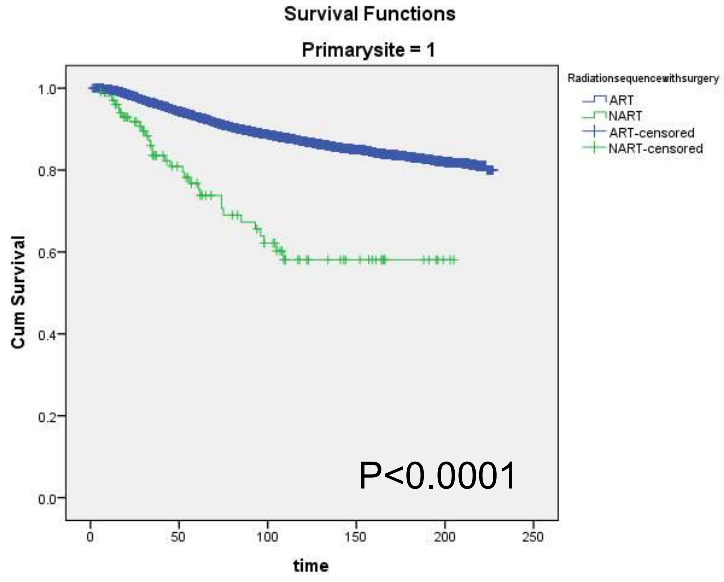

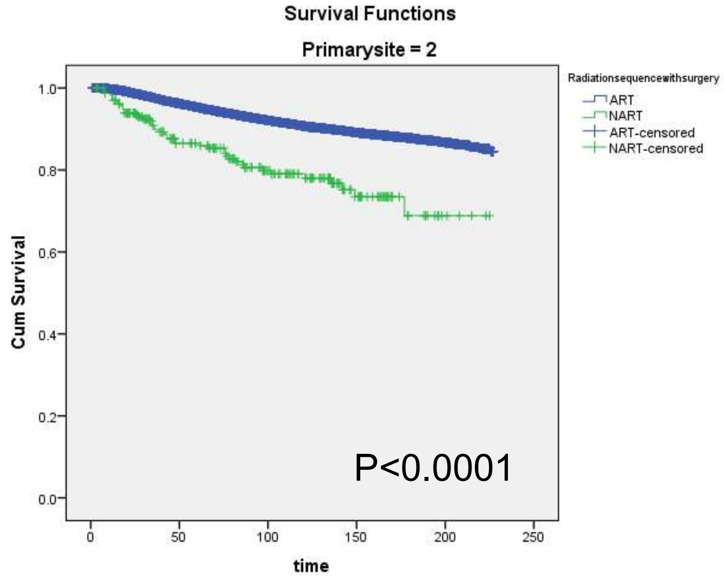

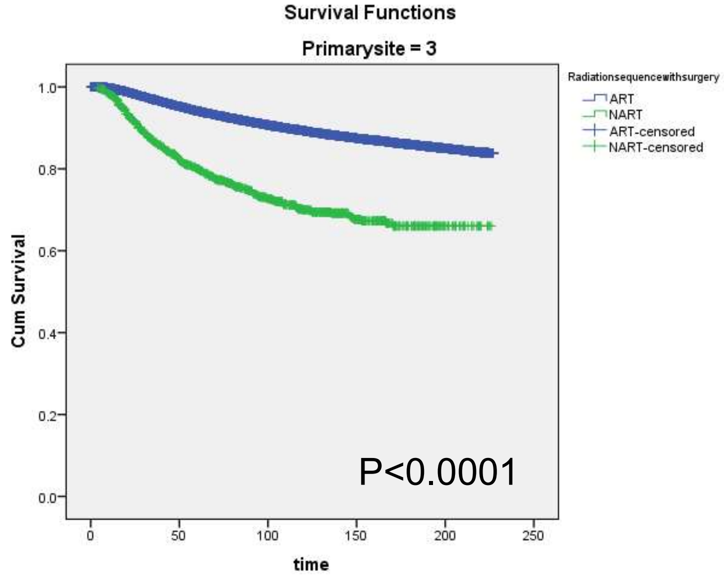

Stratum: Subtype= 1, 1: Luminal A, 2: Luminal B, 3: HER-2 enriched, 4: TNBC, 5: unknown

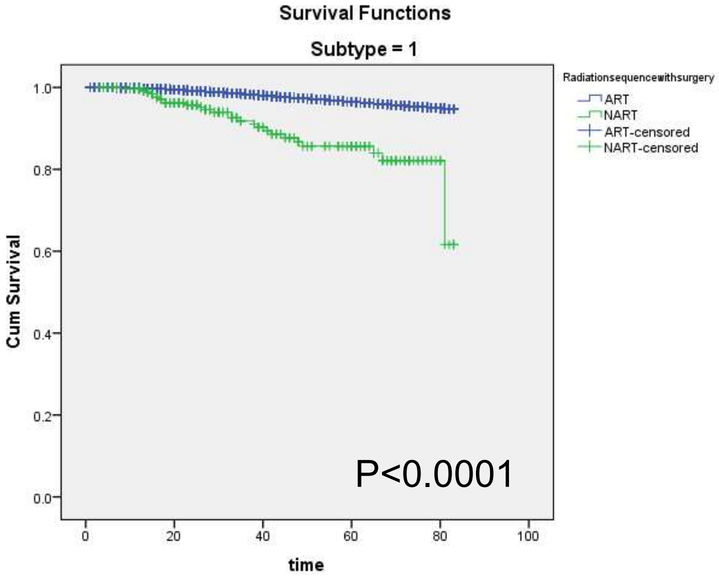

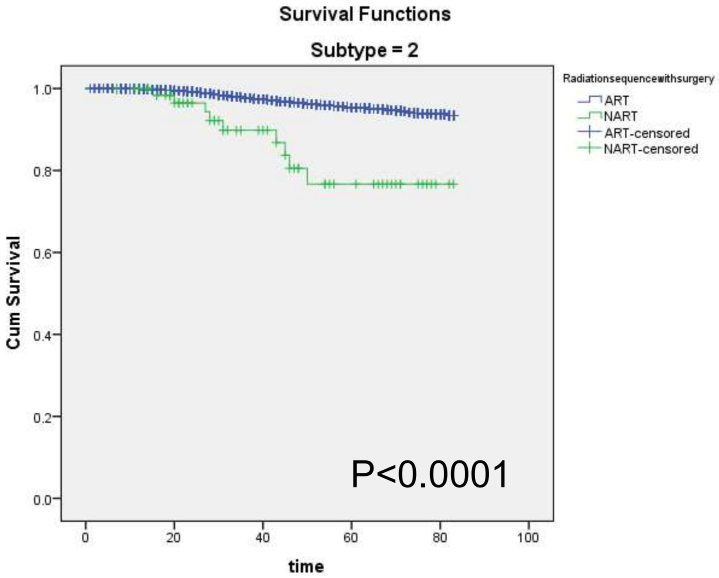

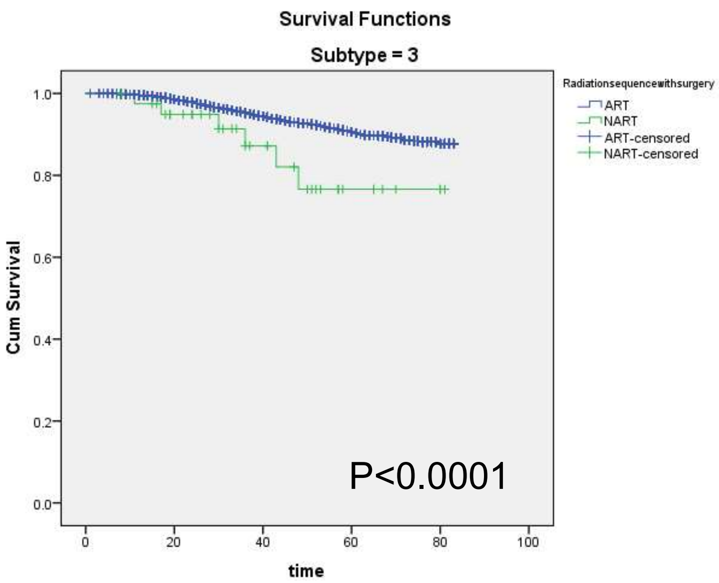
Survival Functions
Subtype = 3
Radiationsequencewithsurgery
ART
NART
ART-censored
NART-censored
Cum Survival
1.0
0.8
0.6
0.4
0.2
0.0
0
20
40
60
80
100
time
P<0.0001

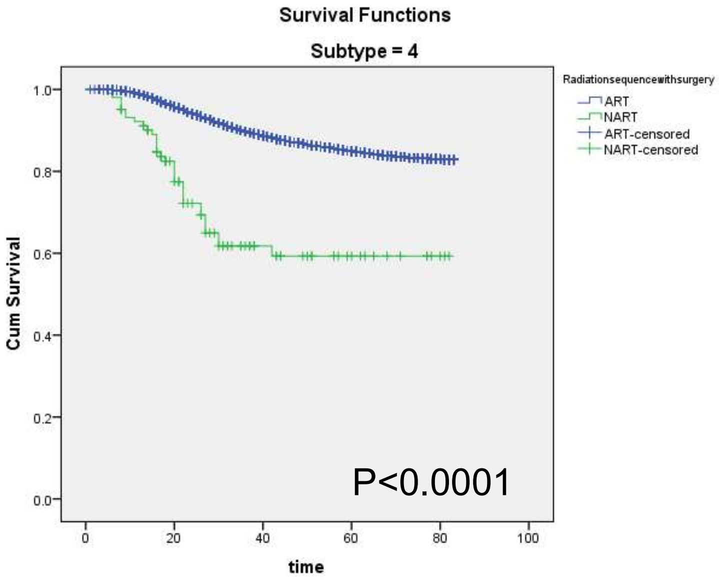
Survival Functions
Subtype = 4
Radiationsequencewithsurgery
ART
NART
ART-censored
NART-censored
Cum Survival
1.0
0.8
0.6
0.4
0.2
0.0
0
20
40
60
80
100
time
P<0.0001

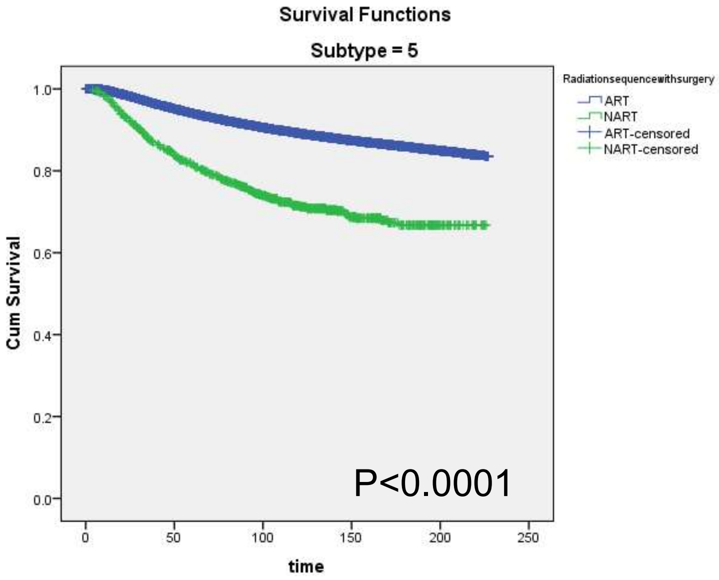
Survival Functions
Subtype = 5
Radiationsequencewithsurgery
ART
NART
ART-censored
NART-censored
Cum Survival
1.0
0.8
0.6
0.4
0.2
0.0
0
50
100
150
200
250
time
P<0.0001

Stratum: Stage, 1: I, 2: II, 3: III

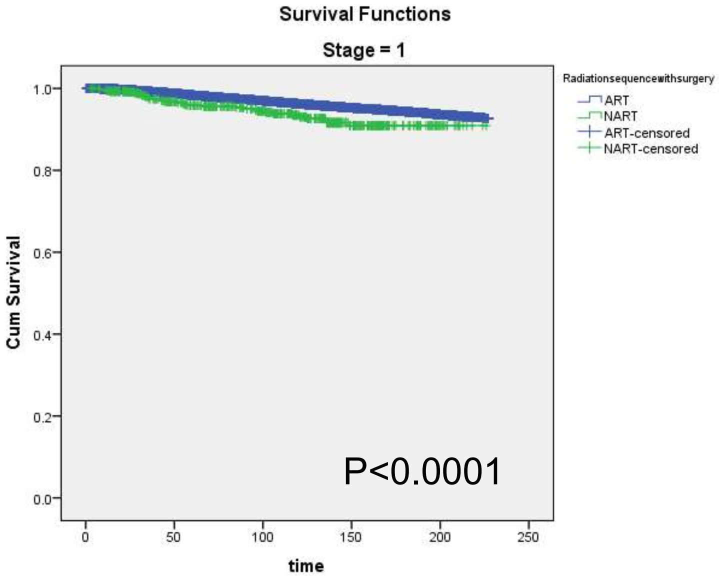

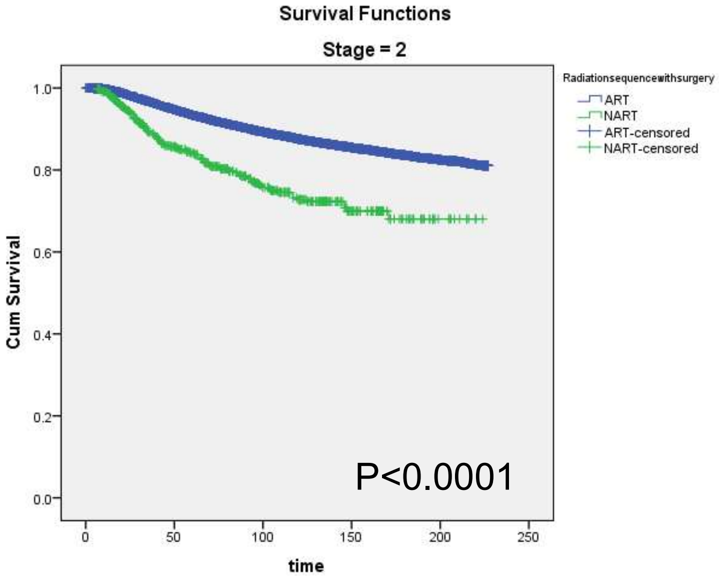

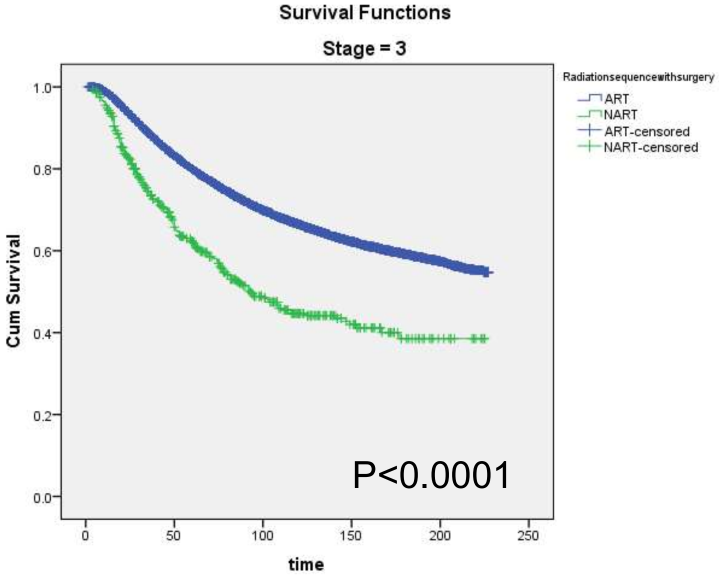

Stratum:Surgery mode, 1: BCS, 2: mastectomy

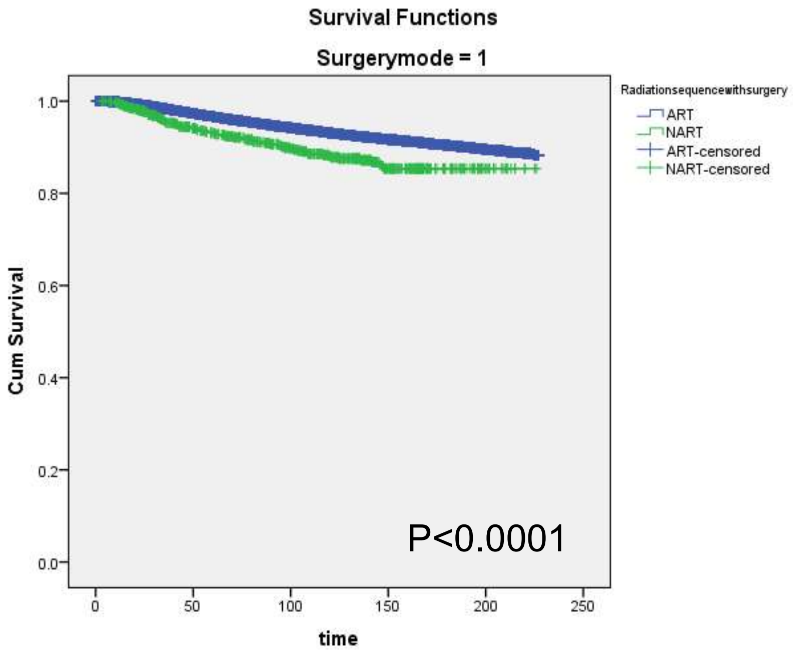

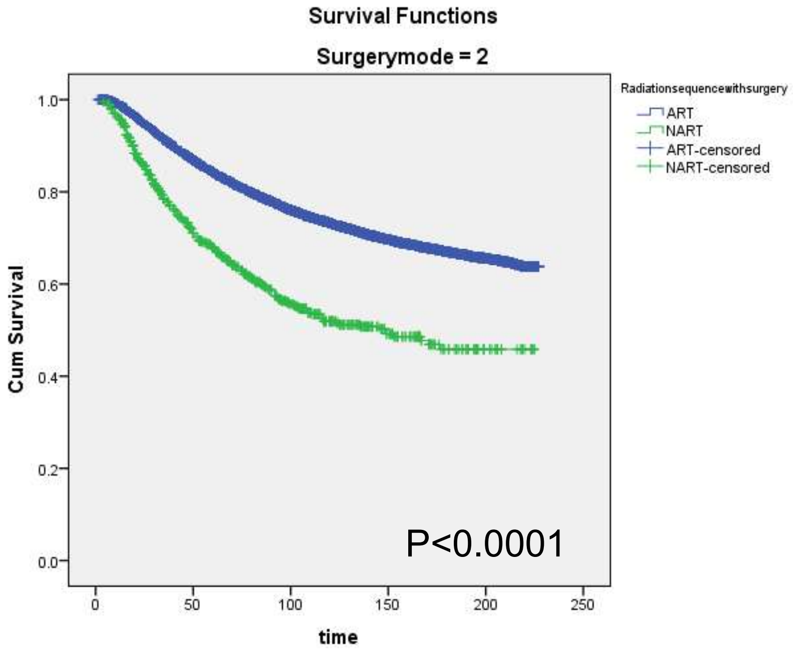

Stratum: Chemotherapy, 1: yes, 2: no/unknown

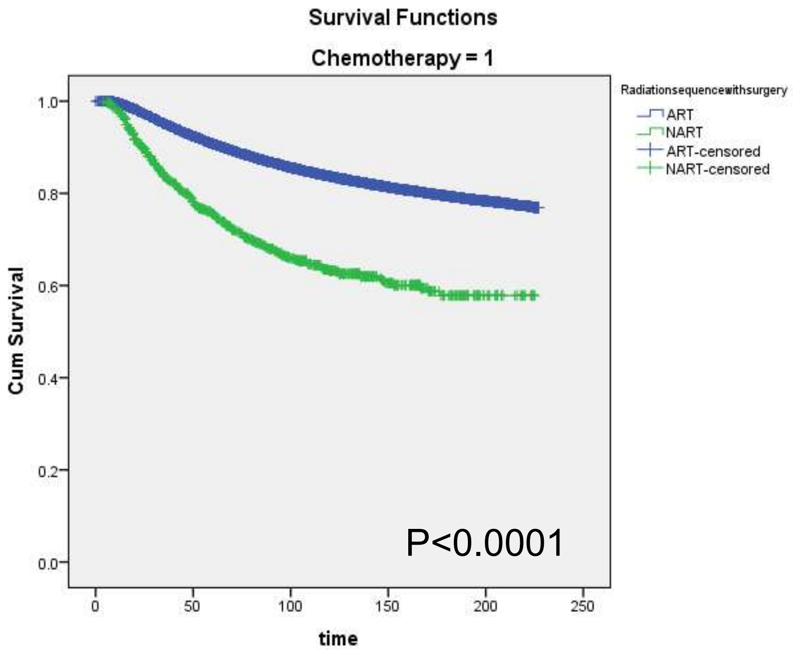

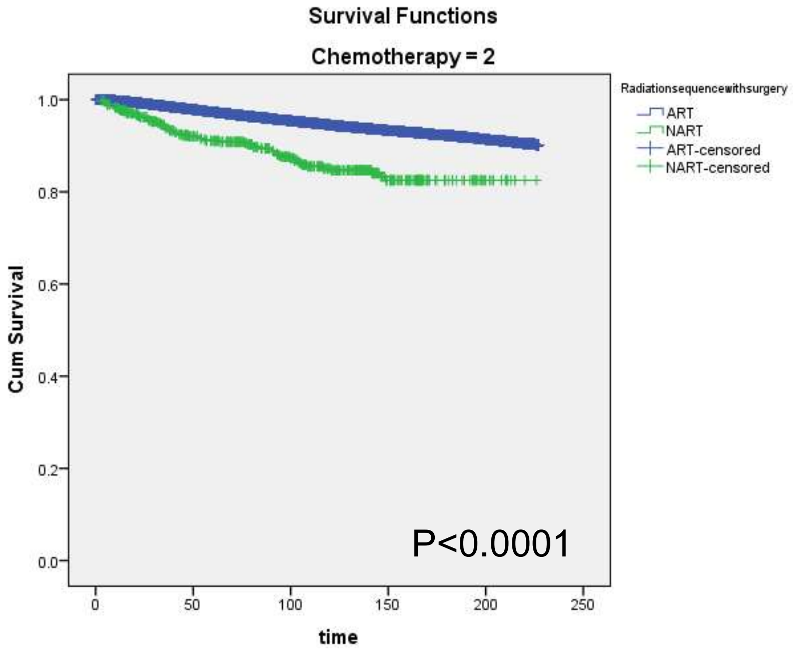

Supplementary Figure 3: Overall breast cancer–specific survival by radiation sequence with surgery among all subgroups.
