# Supplementary material for: Survival comparison between postoperative and preoperative radiotherapy for stage I–III non-inflammatory breast cancer
Source: Sci Rep. 2022 Aug 22;12:14288. doi: 10.1038/s41598-022-18251-3 (PMC9395522; doi:10.1038/s41598-022-18251-3)
Supplement: Supplementary file 4 — Supplementary Figure 4. [file 41598_2022_18251_MOESM4_ESM.pdf]

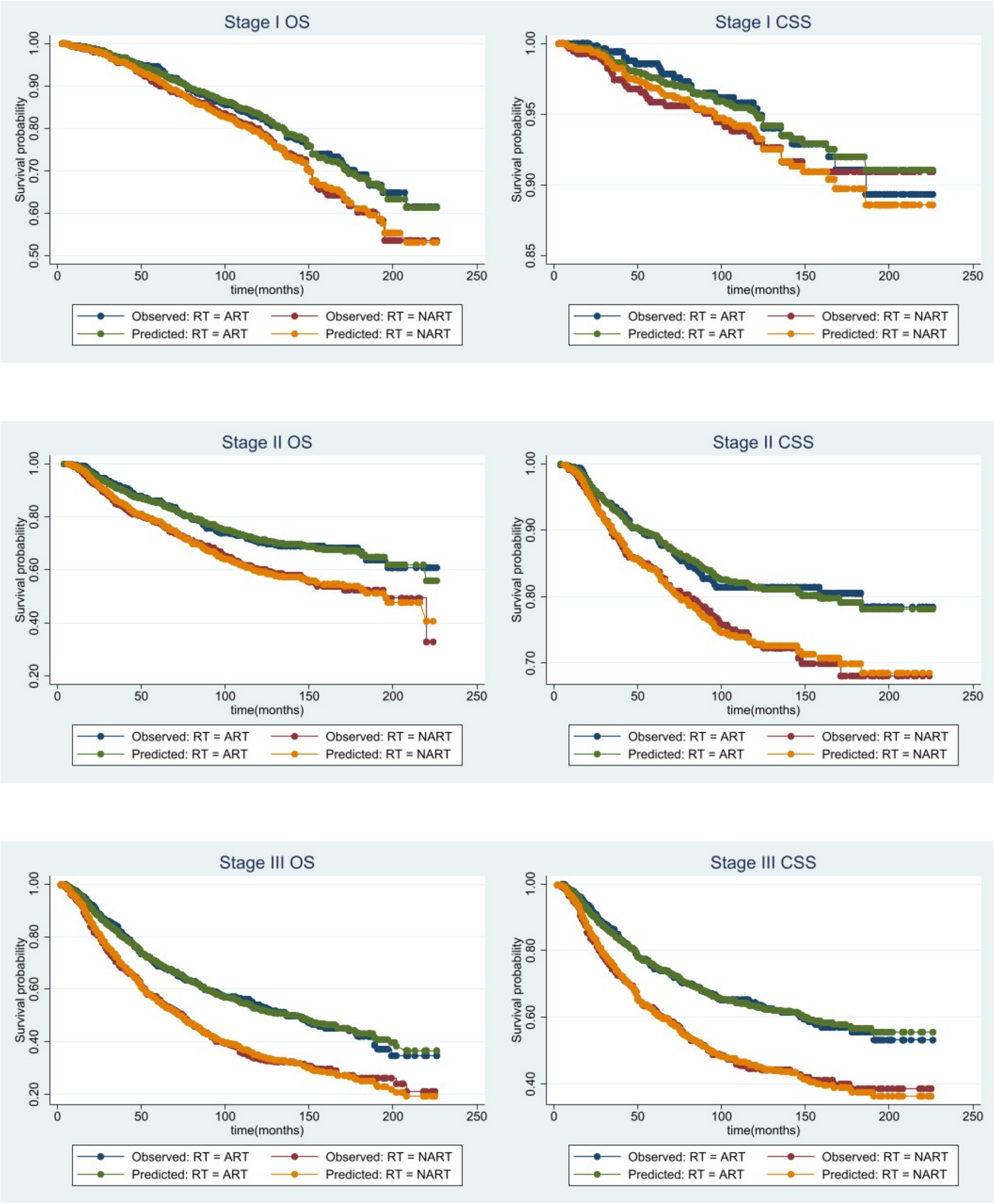

Supplementary Figure 4: Interaction test of overall and breast cancer-specific survival by Cox model including radiation sequence in the stage subgroup based on propensity score matching -adjusted survival data.
