# Supplementary material for: Survival comparison between postoperative and preoperative radiotherapy for stage I–III non-inflammatory breast cancer
Source: Sci Rep. 2022 Aug 22;12:14288. doi: 10.1038/s41598-022-18251-3 (PMC9395522; doi:10.1038/s41598-022-18251-3)
Supplement: Supplementary file 6 — Supplementary Information. [file 41598_2022_18251_MOESM6_ESM.pdf]

Supplementary material. 6 Cox regression of death caused by other reasons between ART and NART group

Supplementary table. 6.1 Case Processing Summary

		N	Percent
Cases available in analysis	Event a	33833	40.9%
	Censored	48975	59. 1%
	Total	82808	100.0%
Cases dropped	Cases with missing values	0	0.0%
	Cases with negative time	0	0.0%
	Censored cases before the earliest event in a stratum	1	0.0%
	Total	1	0.0%
Total		82809	100.0%

a. Dependent Variable: time

Supplementary table. 6.2 Categorical Variable Codings[a]

		Frequency	(1)
Radiation sequence with surgery [b]	ART	82164	1
	NART	645	0

a. Category variable: Radiation sequence with surgery (Radiationsequencewithsurgery)

b. Indicator Parameter Coding

Supplementary table. 6.3 Variables not in the Equation[a]

	Score	df	Sig.
Radiation sequence with surgery	236.781	1	.000

a. Residual Chi Square = 236.781 with 1 df Sig. = .000

Supplementary table. 6.4 Omnibus Tests of Model Coefficients[b]

Step	-2 Log Likelihood	Overall (score)			Change From Previous Step			Change From Previous Block		
		Chi-square	df	Sig.	Chi-square	df	Sig.	Chi-square	df	Sig.
1[a]	718428.735	236.781	1	.000	179.523	1	.000	179.523	1	.000

a. Variable(s) Entered at Step Number 1: Radiation sequence with surgery

b. Beginning Block Number 1 . Method = Forward Stepwise (Likelihood Ratio)

Supplementary table. 6.5 Variables in the Equation

	B	SE	Wald	df	Sig.	Exp(B)	95 .0% CI for Exp(B)	
							Lower	Upper
Step 1 Radiation sequence with surgery	-.750	.050	225.992	1	.000	.472	.428	.521

Supplementary table. 6.6 Model if Term Removed

Term Removed	Loss Chi-square	df	Sig.
Step 1 Radiation sequence with surgery	179.523	1	.000

Supplementary table. 6.7 Covariate Means and Pattern Values

	Mean	Pattern	
		1	2
Radiation sequence with surgery	.992	1.000	.000

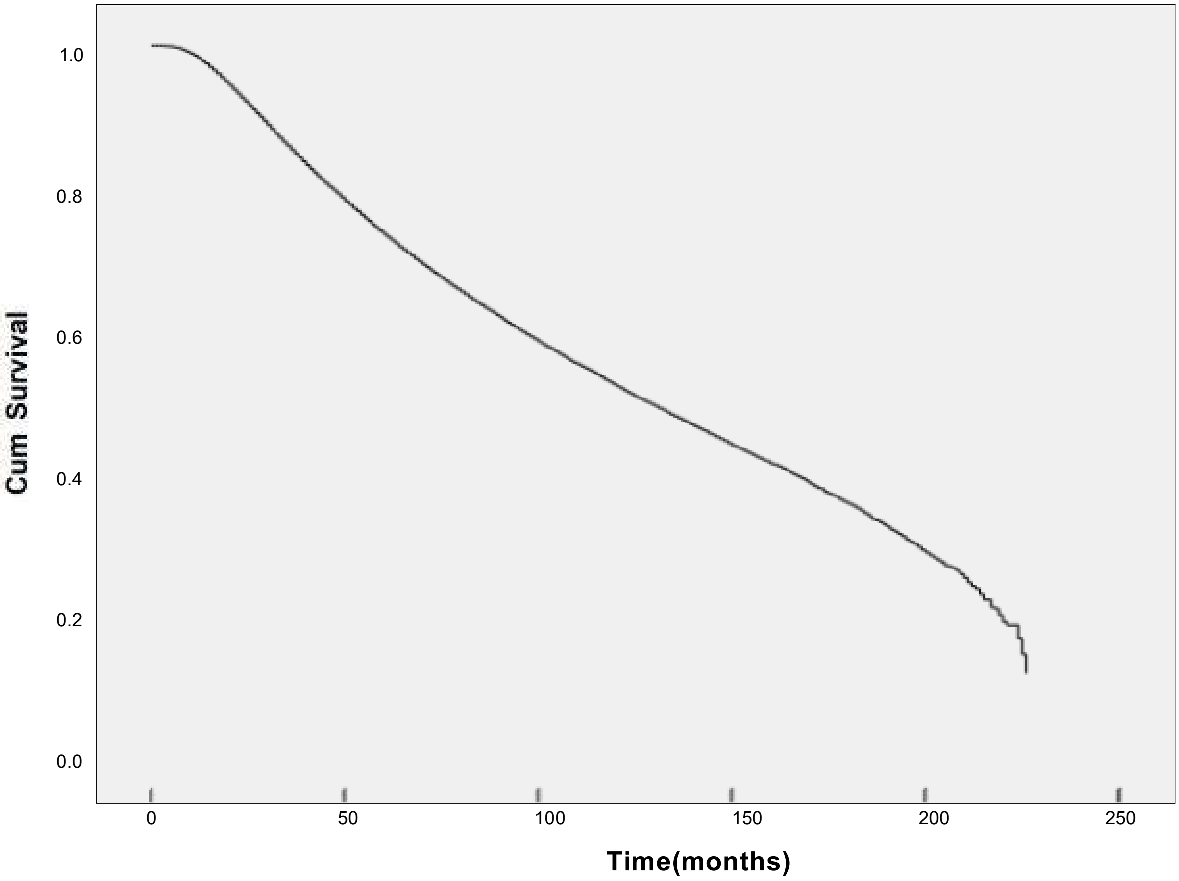

Supplementary figure. 6.1 Survival Function at mean of covariates

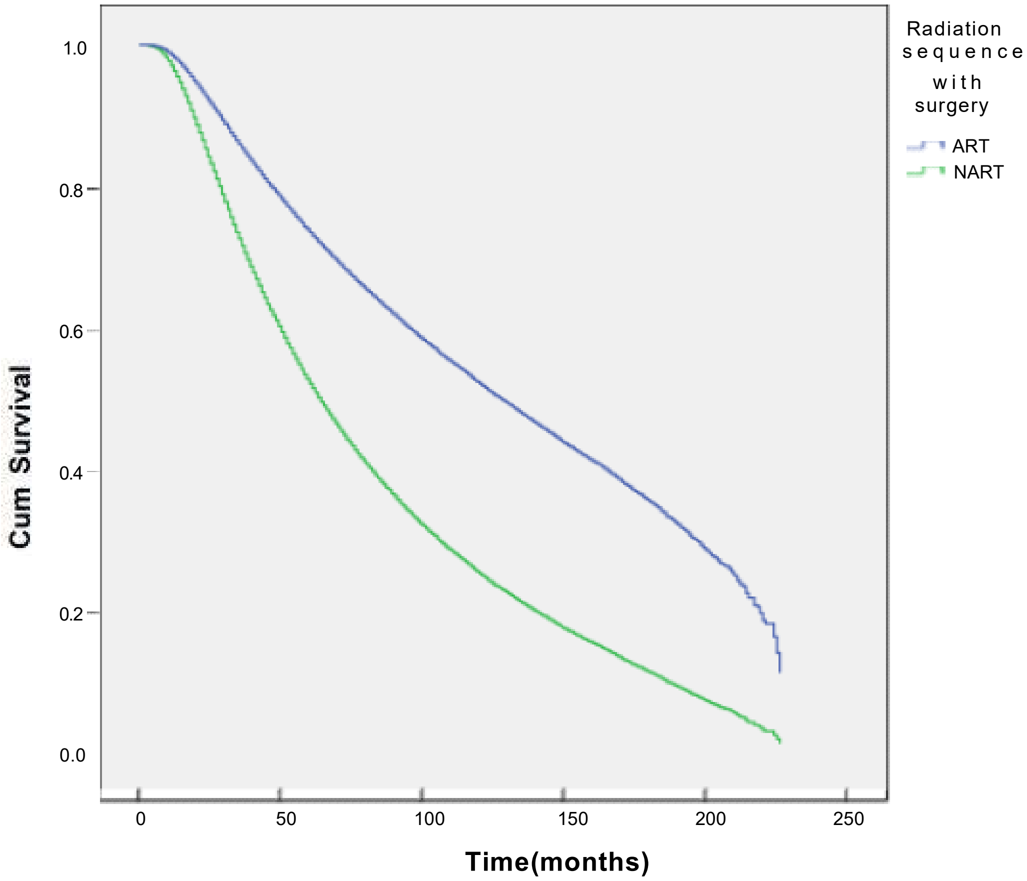

Supplementary figure. 6.2 Survival Function for patterns ART and NART

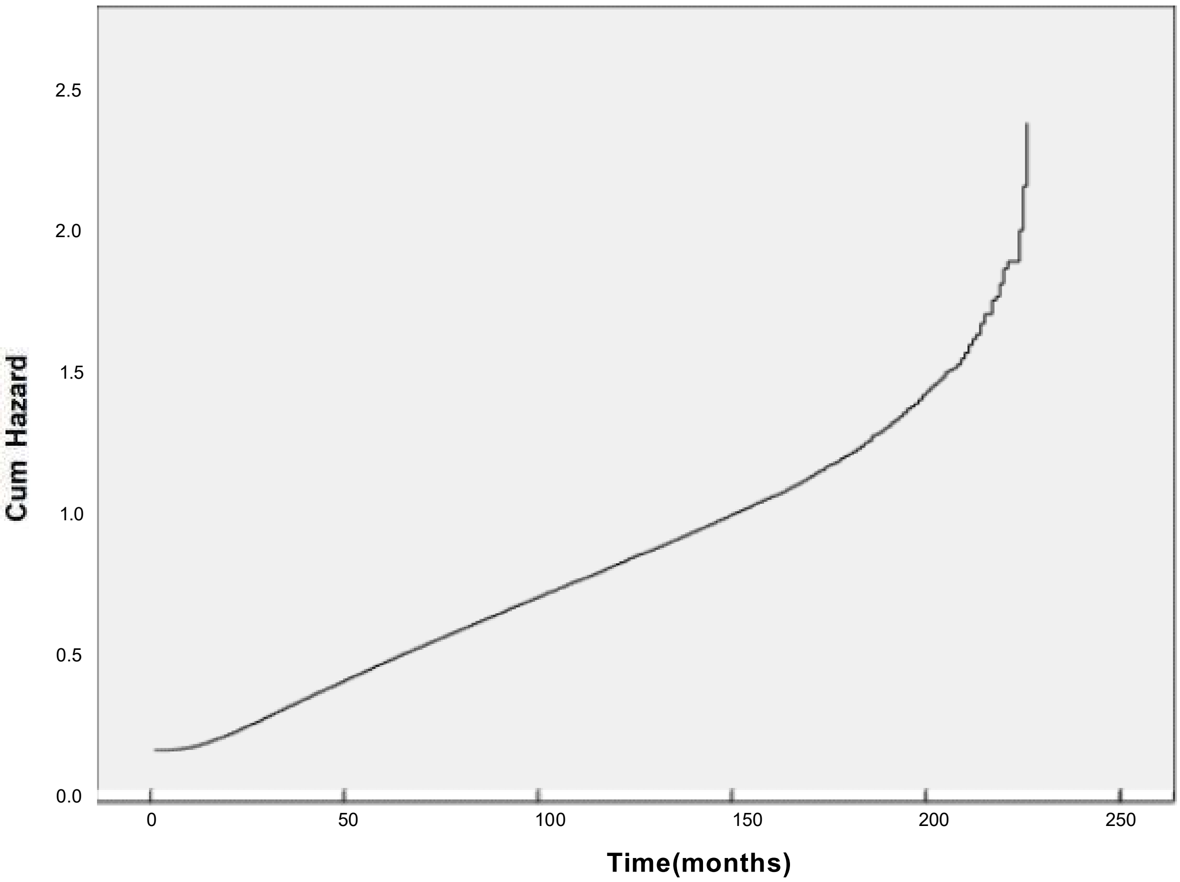

Supplementary figure. 6.3 Hazard Function at mean of covariates

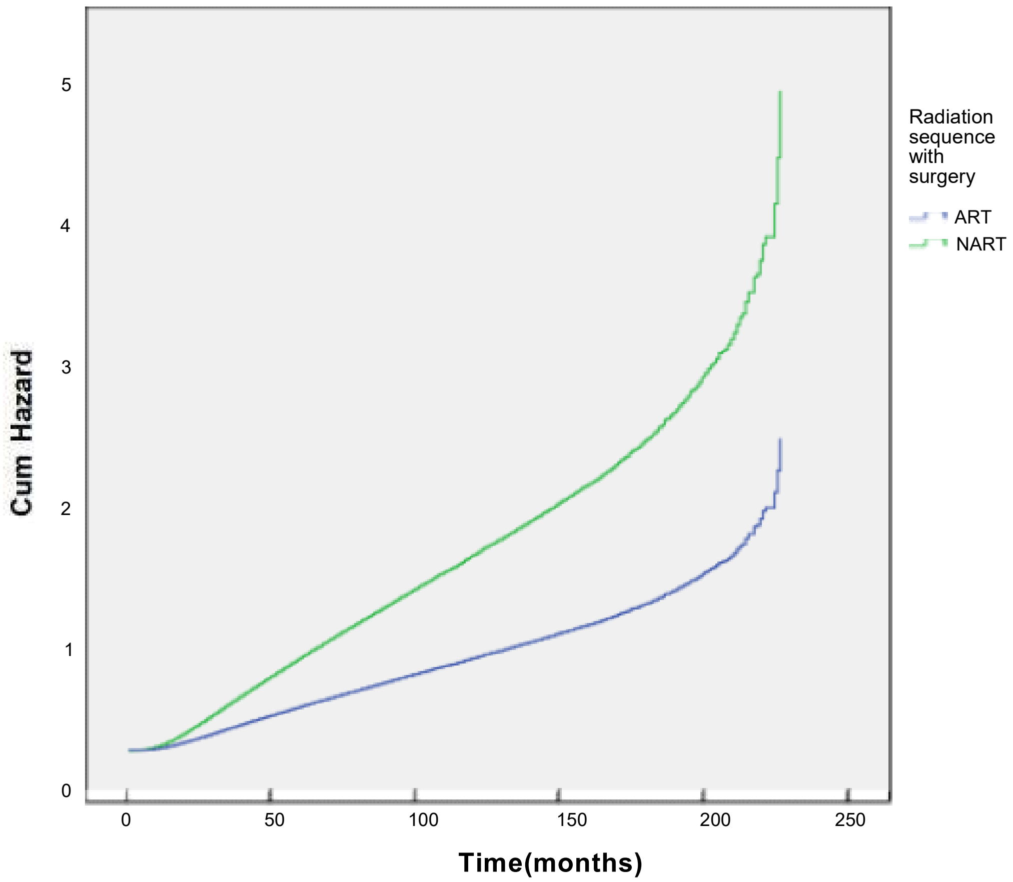

Supplementary figure. 6.4 Hazard Function for patterns ART and NART
